# Image-Analysis-Based Validation of the Mathematical Framework for the Representation of the Travel of an Accelerometer-Based Texture Testing Device

**DOI:** 10.3390/s25206307

**Published:** 2025-10-12

**Authors:** Harald Paulsen, Margit Gföhler, Johannes Peter Schramel, Christian Peham

**Affiliations:** 1 Research Unit for Biomechanics and Rehabilitation Engineering, TU Wien, 1060 Vienna, Austria; margit.gfoehler@tuwien.ac.at; 2 Movement Science Group, University Equine Hospital, University of Veterinary Medicine, 1210 Vienna, Austria; johannes.schramel@vetmeduni.ac.at (J.P.S.); christian.peham@vetmeduni.ac.at (C.P.)

**Keywords:** high-speed recording, image analysis, accelerometer, mathematical models

## Abstract

Texture testing is applied in various industries. Recently, a simple, accelerometer-equipped texture testing device (Surface Tester of Food Resilience; STFR) has been developed, and we elaborated formulae describing the movement of the probe. In this paper, we describe the validation of said formulae, relying on video image analysis of the travel of the spherical probe. This allowed us to select the best-fit mathematical models. We elaborated formulae for accurate calculation of specimen surface characteristics and present an application integrating these formulae in the test procedure. The impact of correct height adjustment and specimen height was found to be critical for reproducibility of measurements and thus needs attendance. These findings form the basis for future comparative studies with established texture analyzers.

## 1. Introduction

The mechanical properties of food are crucial for its texture, processing, and consumer acceptance. There are various methods for testing these properties, depending on the objective (e.g., firmness, elasticity, viscosity), because texture is not only a sensory quality characteristic but also a technologically relevant parameter. Objective measurement of these properties is achieved through a variety of instrumental methods, including compression tests, shear analysis, and rheological measurements, which are increasingly complemented by imaging techniques [1].

In particular, in meat processing, the precise determination of texture—for example, using the Warner–Bratzler shear test [2,3] or Texture Profile Analysis (TPA) [4]—is essential for assessing tenderness, juiciness, and fiber structure. Simultaneously, sensory methods, such as trained sensory panels, provide valuable subjective data that often, albeit not always, correlate with instrumental results [5] and are used for validation [1,6].

Food physics provides a comprehensive theoretical foundation, systematically describing and quantifying the mechanical, thermal, and rheological properties of food. Modern developments in process automation and the use of online sensors enable continuous quality control throughout the entire production chain [1].

As regards meat and meat products, Warner-Bratzler shear tests and Texture Profile Analysis (TPA) are still widely used in quality control and product development. The Warner–Bratzler method reports force–distance data for shearing a specimen with defined diameter and can give information not only on the maximum force needed to shear the specimen but also on its fracturability/brittleness. Similarly, TPA reports force–distance data, but for two consecutive compression–relaxation cycles. From the maximum forces and the areas under the curves (“work”), some material characteristic can be derived [7]. For example, the hardness is the maximum force during compression; the resilience is the ratio of the upstroke energy of the first compression by the downstroke energy of the first compression [7]. Data from both compression–relaxation cycles can be used to calculate characteristics as chewiness, springiness, etc., related to the sensations consumers experience during chewing foods, thus the colloquial name “two-bite test” for TPA.

Yet, such devices are costly and not portable. Likewise, maintaining a panel of trained testers is often not feasible. Arguably, the provision of a small, inexpensive and easy-to-operate texture tester would allow also smaller food businesses to examine the textural quality of their products.

Recently, an accelerometer-equipped texture testing device (Vienna Surface Tester (VST)) has been developed [8]. A scaled-down version (Surface Tester of Food Resilience (STFR)) of said device has been proposed as a food texture tester. Whereas the original device operates in free fall, the probe of the scaled-down device follows a circular, arc-shaped path. Both devices use a sphere equipped with accelerometers. The sphere is dropped onto the specimen’s surface and eventually bounces back. Changes in acceleration over the measurement duration are recorded (see Section 2.1) and some material characteristic values are calculated, e.g., the spring constant, Young’s Modulus, and Energy Recovery [8].

As regards shear force, a preliminary study showed correlations for shear force (Warner–Bratzler) with spring constant (STFR), Young’s Modulus, and Resonance Frequency for solid foods [9], albeit for drop heights of 25 mm and 50 mm only, and with a less refined rig and software.

However, for TPA, such a comparison is lacking. This was mainly due to the finding that the current mode of data processing in the STFR is inaccurate at dropping heights exceeding 17 mm [10]. This is due to the fact that the calculations performed by the current version of the STFR tester rely on a free-fall model.

In order to obtain correct data for other drop heights, formulae describing the movement of the sphere have been elaborated [10], based on a hammer, or a free fall/hammer average model instead of a free-fall-only model.

The aim of this article is to compare the outcomes of said formulae with raw data retrieved from the device and from image analysis of high-speed video recordings in order to select the most appropriate formulae for the sphere’s movement; and, second, to derive formulae for material characteristics from these identified formulae.

These formulae will allow a thorough comparison of the STFR-generated results for spring constant and penetration depth to TPA generated values for Hardness. Likewise, we assume a relation of Energy Recovery (STFR) with Resilience (TPA). For a comparison of TPA variables relying on two compression-relaxation-cycles, the formulae presented in our manuscript will be simply applied on the first and on the second drop-bounce-cycle.

In Section 2.1 and Section 3.1, we describe the principle of operation and the parameters to be validated. Section 2.2 describes statistical procedures and software used. In Section 2.3, the setup for kinematic analysis of the travel of the STFR probe by high-speed image analysis is detailed. Section 2.4 defines the variables considered for comparison of model-generated data with STFR and image analysis, whereas in Section 3.2, Section 3.3, Section 3.4 and Section 3.5, the outcomes of said comparisons are presented, resulting in identifying the best-matching formulae among the set specified in [10]. Section 3.6 summarizes the appropriate formula for characterizing material properties. Section 2.5 and Section 3.7 present the design of an MS-Access^®^-based data processing and recording application.

## 2. Materials and Methods

### 2.1. Device and Principle of Operation

**Figure 1:**
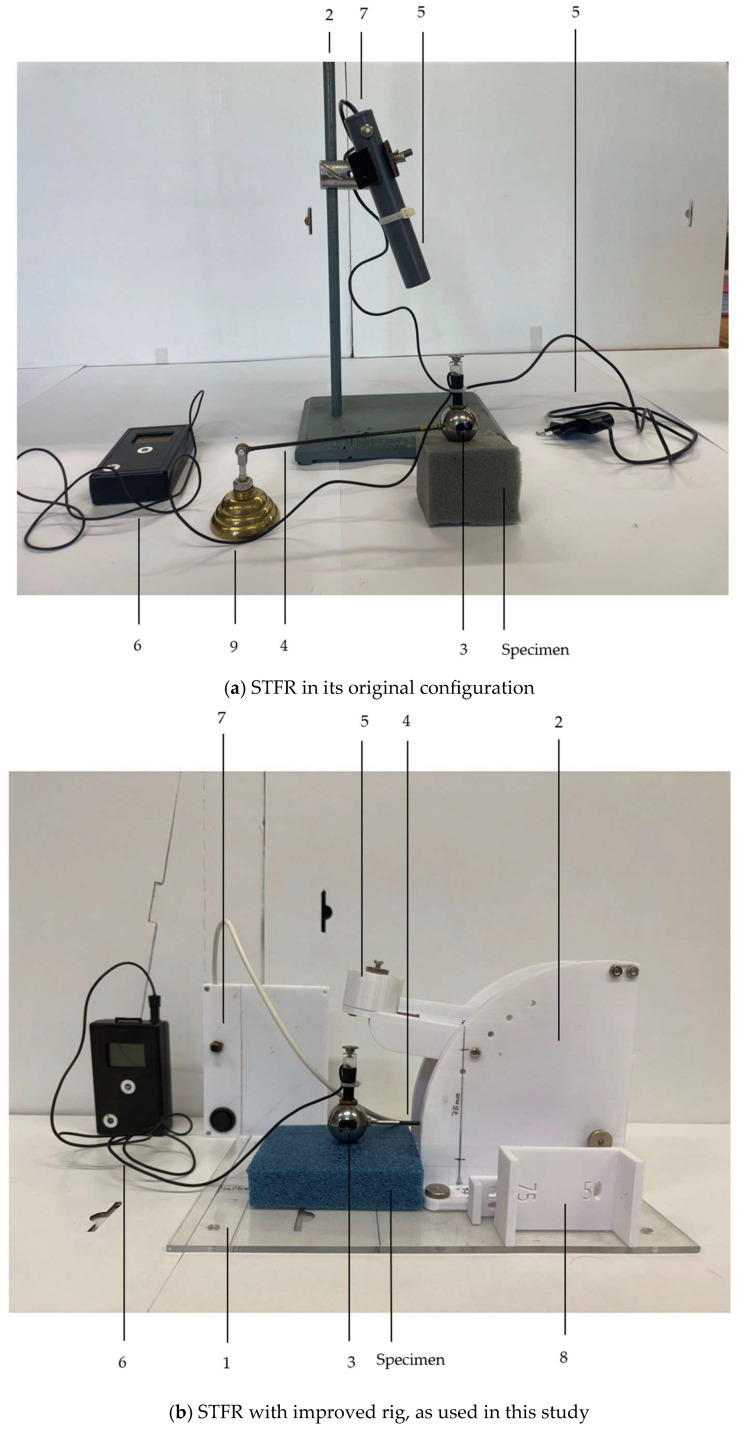
Main components of the STFR. Figure (**a**) shows the initial version of the prototype of 2015. The height is adjusted by positioning the magnetic trigger unit on a rig. Figure (**b**) shows a rig with pre-defined heights and a magnetic mount ensuring full contact of the magnetic coupling in all starting positions (1 = baseplate; 2 = rig with an electromagnet adjustable in height; 3 = sphere with two built-in accelerometers; 4 = rod (carbon fiber) connecting the sphere to a swivel; 5 = the sphere’s contact surface for the electromagnet device; 6 = digital data acquisition system; 7 = trigger for the electromagnetic holder and power supply; 8 = gauges (25 mm, 50 mm, 75 mm) to check the distance between specimen surface and sphere; 9 = base for the swivel).

The main components of the STFR are a spherical probe, which is attached to a swivel via a carbon-fiber rod, a data-recording and -processing unit and a magnetic release mechanism (Figure 1). A prototype (version 2015, Vienna, Austria) with electromagnetic release is shown in Figure 1a, whereas a more rugged design (used from 2023 onwards) allowing more reproducible adjustments is depicted in Figure 1b.

Before measurement, the probe is positioned at a predefined height above the specimen and held by an electromagnet. By breaking the circuit of the electromagnet, the probe is released and falls along a circular trajectory until touching the specimen. Depending on the nature of the specimen, the probe will indent the surface of the specimen to some extent and bounce back. The cycle of downward and upward travel is repeated, albeit with decreasing amplitude, until the probe comes to rest on the specimen’s surface.

Since the circuit remains interrupted until the next measurement, no interference with magnetic forces is expected.

During the movement of the sphere, acceleration is sensed by two built-in accelerometers and time and changes in speed are recorded [8]. Table 1 lists the variables recorded by the STFR and their units.

**Figure 2:**
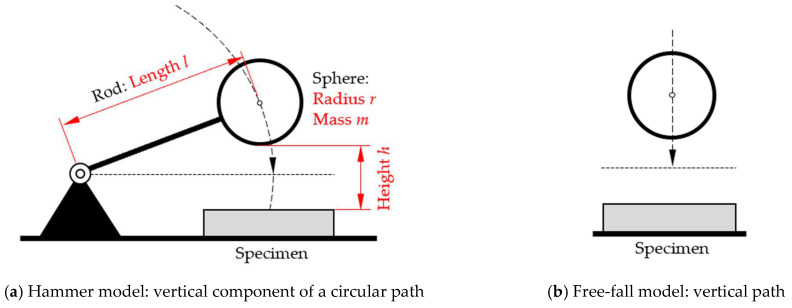
Modeling the movement of the sphere: (**a**) setting for the hammer model and associated model parameters; (**b**) free-fall model (illustration not to scale).

Based on these recorded data, we elaborated formula sets to calculate the surface characterization parameters. The formula sets are based on mathematical-physical models developed in a previous study [10]. These models either consider the circular path of the sphere, like the movement of a hammerhead (“hammer model”, Figure 2a), or assume a vertical travel (“free-fall model”, Figure 2b) or use an arithmetic average of the results of both models (“average model”). The derivation of the models is described in [10]. For any calculation in this study, we used mass of the sphere *m* = (0.104 ± 0.001) kg, length of the rod *l* = (170 ± 1) mm, and gravitational acceleration *g* = 9.81 m·s^−2^.

### 2.2. Computational Procedures and Statistics

Per experimental settings, five replicate measurements were made. Mean value and standard error were calculated with Microsoft Excel^®^ V. 2406 Microsoft 365 for Enterprise. The standard error was calculated as $SE=\frac{s}{\sqrt{n}}$, where *s* = standard deviation and *n* = number of replicate measurements.

Values calculated using formulas are given with their maximum error. For a given formula, for example, f=f(x,y) and a point $x=x_{0}\pm ∆x,\;y=y_{0}\pm ∆y$, the function value $z=z_{0}\pm ∆z$ is calculated as $z_{0}=f(x_{0},y_{0})$ and its maximum error as $∆z={\left|\frac{\partial f}{\partial x}\right|}_{x=x_{0},y=y_{0}}\cdot ∆x+{\left|\frac{\partial f}{\partial y}\right|}_{x=x_{0},y=y_{0}}\cdot ∆y$. The formulas and error calculations used in this article were derived in [8,10] and File S2. The calculations are performed with PTC Mathcad Prime 10.0.1.0.

The movement data (times and heights) determined using the Kinovea 0.9.5 software (www.kinovea.org (accessed on 2 June 2024)) are further processed in Microsoft Excel^®^.

### 2.3. Setup for the Kinematic Analysis

#### 2.3.1. Placement of the Camera Relative to the STFR

The travel of the sphere was recorded with a high-speed camera (Sony RX100 M4; Sony, Tokyo, Japan). The camera axis was the perpendicular left side of the STFR. The distance of the front of the camera objective to the center of the sphere was 365 mm, and the center of the lens and the center of the sphere were aligned on a horizontal axis. Since the distance from lens to sphere is large compared to the diameter of the sphere, perspective distortion was not considered.

#### 2.3.2. Setup of the STFR to Determine the Influence of the Initial Position of the Sphere

We studied if and how the falling time *T*_0_ is affected when the initial position of the sphere is changed. To this end, thickness of the specimen was set to 25 mm, and the starting position was adjusted to give a distance ∆*x* between the top surface of the specimen to the lowest part of the sphere of 25 mm and to ensure that the rod is in horizontal position when the sphere impacts the specimen.

**Figure 3:**
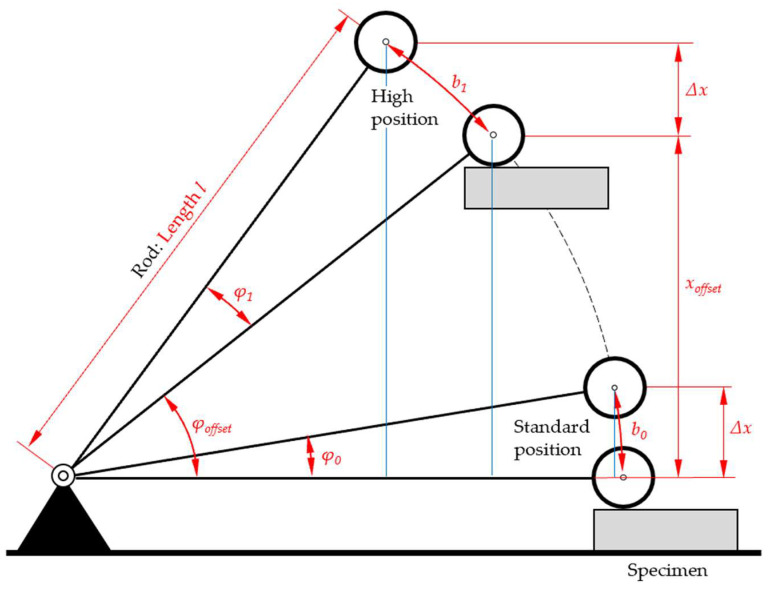
Diagram of the STFR, with same initial sphere-to-specimen distance but different elevation angle to the horizontal line. In the standard position, the rod is vertical when the sphere hits the specimen, with an elevation angle *φ*_0_. In “high position” (vertical distance *x_offset_*), with the same sphere–specimen distance ∆*x*, the elevation angle of *φ*_1_ differs from *φ*_0_ (illustration not to scale).

Any change in the initial height (“high position”) of the sphere corresponds to a change in the elevation angle of the rod and will result in an inclined position of the rod at the time of impact of the sphere, represented by the angle *φ_offset_*; see Figure 3.

**Figure 4:**
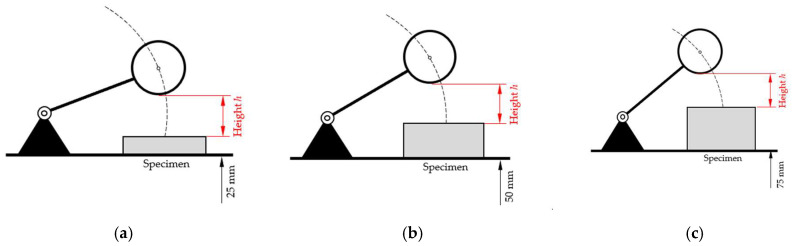
Configurations for different distance of the specimen’s top surface to the baseplate but identical difference from specimen’s surface (*h* = 25 mm). (**a**) Standard configuration with 25 mm and *x_offset_* = 0 mm. Elevated position of the sphere with specimen thickness of 50 mm (**b**) and 75 mm (**c**) (illustration not to scale).

The effect of *φ_offset_* on the time to impact *T*_0_ was assessed experimentally. The specimen’s surface was positioned in different heights relative to the baseplate (Figure 4).

**Figure 5:**
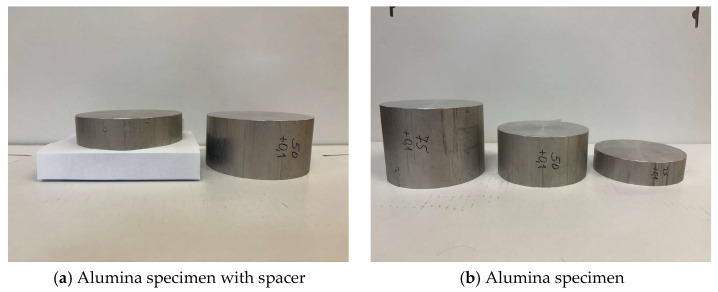
Specimens used for experiments with different distance of the specimen’s top surface to the baseplate: (**a**) comparison of an alumina specimen with 25 mm thickness placed on a socket of same thickness with an alumina specimen of 50 mm height; (**b**) specimens under study: alumina cylinders of 100 mm diameter and 25 mm, 50 mm, and 75 mm thickness.

Originally, we wanted to use cylindrical alumina specimens with a diameter of 100 mm and a thickness of 25 mm in combination with bases with a thickness of 25 mm and 50 mm (Figure 5a), but ultimately we used solid specimens with a thickness of 25 mm, 50 mm, and 75 mm (Figure 5b). This was based on the consideration that the mass of the sphere and the distance to contact with the specimen (25 mm) would not deform the alumina surface, regardless of the thickness being 25 mm or more than 25 mm.

#### 2.3.3. Setup of the STFR for Comparison with Model Data and Kinematic Analyses

Data generated by these measurements serve to assess the models presented in [10]. Five measurements were made with 25 mm, 50 mm, and 75 mm initial height (±1 mm) on a foam board (=specimen) with 100 mm × 100 mm base area and 25 mm thickness. The travel of the sphere was recorded with a high-speed camera (see Section 2.3.1).

#### 2.3.4. Kinematic Analysis of the Travel of the Sphere

The arrangement of the camera was as described in Section 2.3.1. The camera recorded for 2 s. Time–position data of the sphere are generated by Kinovea 0.9.5. software [11]. The following arrangements were made:

- The capture–frame-rate was set to 1000 fps (frames per second).
- The sphere is placed on the specimen (height *h* = 0 mm), and the axis of view is horizontal. The center of the apparent outline of the sphere is marked.
- The path of the mark is recorded by Kinovea.
- The vertical positions ([px]) and time ([ms]) are exported in a .csv-file.

In order to allow comparison of mathematical models with data from the video image analysis, the latter were adapted using Microsoft Excel^®^:

- Vertical positions were smoothed by a simple-moving-average (SMA) filter [12].
- For each measurement, the starting height *h_start_* was calculated as the average of the first 100 data points, and the end height *h_end_* was calculated as the average of the last 100 data points.
- For each measurement, a conversion factor $cf=\frac{h_{start}-h_{end}}{h+1}$ from pixel [px] to [mm] is determined based on the preadjusted initial height *h* and an assumed indentation of 1 mm of the specimen when hit by the sphere. Five measurements were taken for each of the initial heights of 25 mm, 50 mm, and 75 mm, and mean values and standard errors were calculated from the individual conversion factors.

**Figure 6:**
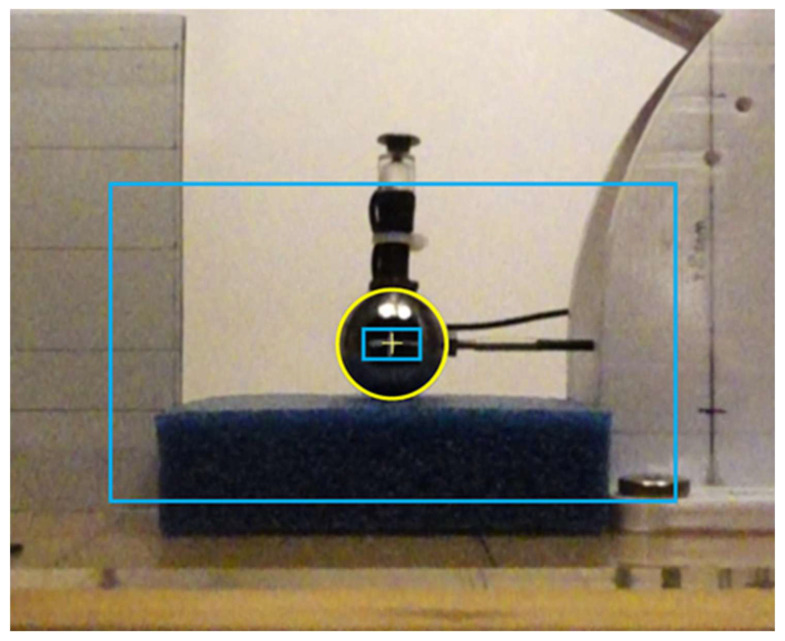
Setting the center mark of the sphere to allow video analysis of the sphere’s movement.

The software would identify a mark placed in the center of the object (see “+” mark in Figure 6). Since this was not always correctly identified, we used the apparent outline as a mark for the sphere (yellow circle in Figure 6). The apparent outline was marked when the sphere first came into contact with the specimen.

### 2.4. Variables Considered for Comparison of Model-Generated Data with STFR and Image Analysis

We checked the quality of the models by comparing results generated by the mathematical models elaborated in [10] with measurements taken by the STFR and a computer-aided analysis of the frames of the video recording. Calculations were performed using Microsoft Excel^®^. All measurements are replicated (*n* = 5, unless stated otherwise). All measured values are reported as averages and standard errors. All calculated values are reported with the maximum error.

For comparison of models and STFR measurements, we used the following variables:

- Time points recorded (*T*_0_, *T*_1_, *d*_0_, *t_P_*_0_);
- Calculated initial height (*h*);
- Depth of penetration (*D = h_min_*);
- Maximum rebound height (*h_max_)*;
- Energy restitution (*E_R_*).

In addition, values for spring constant *k* and damping constant *c* were calculated. For these variables, no reference data can be derived from measurements and image analyses.

In order to identify measurements with the actual height *h* deviating from the prearranged setting, indicative for errors in height adjustment, we elaborated reference values for the time *T*_0_ from release of the sphere to first contact with the specimen.

### 2.5. MS-Access^®^-Based User Interface

This application was designed to processes data generated by the STFR and should allow import of *.csv data, arrangement of results according to date and initial height, setting of tolerance levels, entering specimen information, presenting average results from replicate tests, and, finally, exporting data in *.xls and *.pdf formats.

## 3. Results

### 3.1. Representation of Different Phases of the Sphere’s Travel by High-Speed Image Recording

**Figure 7:**
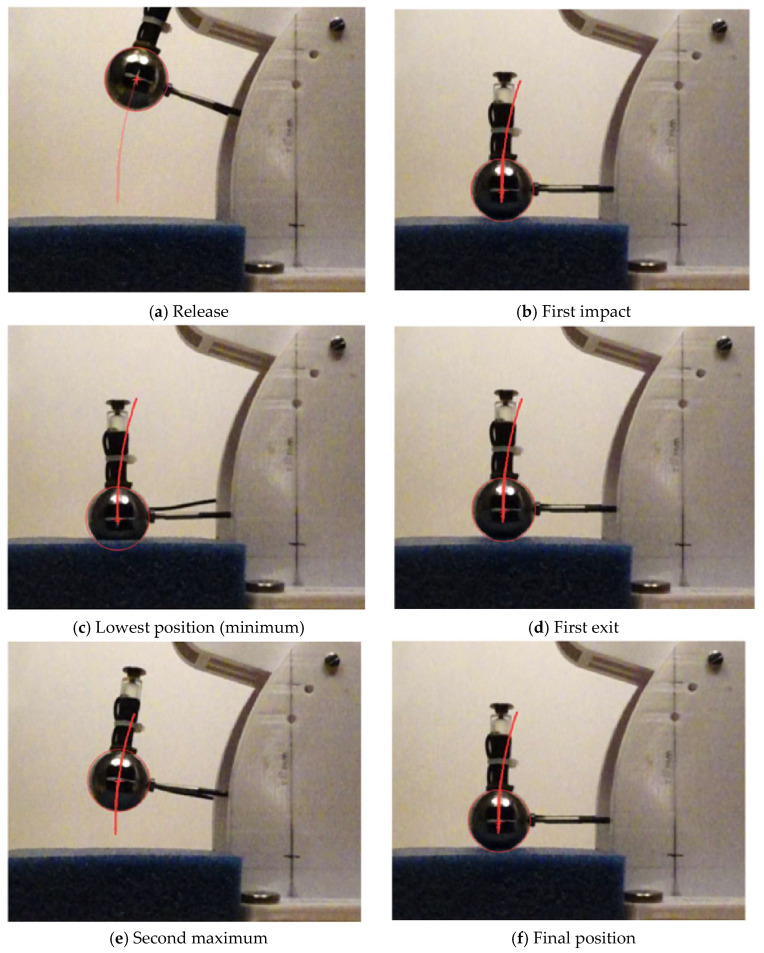
Frames from a high-speed recording of the travel of the sphere. The travel starts from the release position (**a**); at impact (**b**), the sphere has first contact to the specimen’s surface; in the lowest position (**c**), the maximum acceleration *G_max_* is recorded. After the first exit (**d**), the probe reaches the second maximum (**e**), with the height *h_max_*, allowing us to calculate energy restitution. After some cycles, the sphere comes to rest in its final position (**f**). When the specimen is not a rigid body, the sphere will indent the specimen’s surface, and the lowest position of the probe will be lower than at impact position (**b**).

The arrangement of the camera relative to the sphere proved to be effective in generating frames with appropriate resolution. Figure 7 shows frames taken at timepoints when acceleration of the sphere changes.

**Figure 8:**
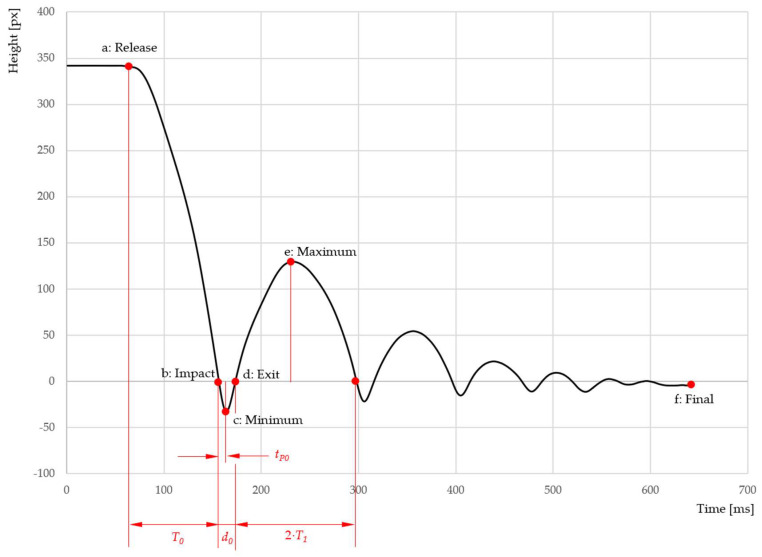
Time–distance plot of the vertical travel of the sphere: time points recorded by STFR and selected points a to f (see also Figure 8) along the trajectory are marked and labeled. The units milliseconds [ms] and pixels [px] shown in the figure are used by the data-collecting software [11]. Time measurement starts when arming the recording unit, whereas the actual travel of the sphere is recorded when the sphere is released from its magnetic holder. The diagram was constructed from the original data using Microsoft Excel^®^.

Figure 8 shows the travel of the STFR’s sphere by plotting distance against time.

### 3.2. Measurement of the Time T_0_ from Release of the Sphere to First Contact with the Specimen

We studied if and how the falling time *T*_0_ is affected when the initial position of the sphere is changed. To this end, thickness of the specimen was set to 25 mm, and the starting position was adjusted to give a distance ∆*x* between the top surface of the specimen to the lowest part of the sphere of 25 mm and to ensure that the rod is in horizontal position when the sphere impacts the specimen (Figure 4). Per setting, five replicate measurements were performed and results are reported in Table 2. Data and corresponding calculations are given in File S1.

Experimental results (Table 2) indicate that the distance of the specimen’s top surface to the baseplate has a non-negligible influence on the time to impact *T*_0_.

A more detailed analysis is performed by mathematical procedures. We compared the STFR’s standard position, with the rod in horizontal position at impact of the sphere, and the “high position”, where the impact to the sphere is at *x_offset_* (Figure 4). We compare *φ*_1_ and *φ*_0_, with the same sphere-to-specimen surface distance and determine the relative deviation *rd_φ_* (Table 3).

This *rd_φ_* is calculated according to Equation (1). The derivation of the formula is given in File S2.

(1) $${rd}_{\varphi }=100\cdot \frac{\varphi_{1}-\varphi_{0}}{\varphi_{0}}=100\cdot \left(\frac{arcsin\frac{∆x+x_{offset}}{l}-arcsin\frac{x_{offset}}{l}}{arcsin\frac{∆x}{l}}-1\right)$$

Due to $b_{0}=\varphi_{0}\cdot l,\;b_{1}=\varphi_{1}\cdot l$, the relative deviation of the arc *b*_1_ to arc *b*_0_ is the same as the relative deviation of the angles *φ*_1_ von *φ*_0_. This results in Equation (2).

(2) $${rd}_{b}={rd}_{\varphi }$$

The time to impact *T*_0_ can be influenced by other factors, such as the friction in the swivel, tangential forces, different magnitude of the tangential force, and different contact points on the sphere’s and the specimen’s surfaces.

### 3.3. Determination of Reference Values for the Time T_0_ from Release of the Sphere to First Contact with the Specimen

The time to impact *T*_0_ is used to assess the validity of a measurement [10]. We now generate reference values for *T*_0_ for a specimen of 25 mm thickness and vertical travels of 25 mm, 50 mm and 75 mm. The specimen is a cylindrical alumina body of 100 mm diameter and 25 mm thickness (Section 2.3.2). Table 4 gives mean values and standard errors for *T*_0_ from five replicates per condition. These reference values will then be termed *T*_0,*ref*_. Calculations are given in File S1.

**Figure 9:**
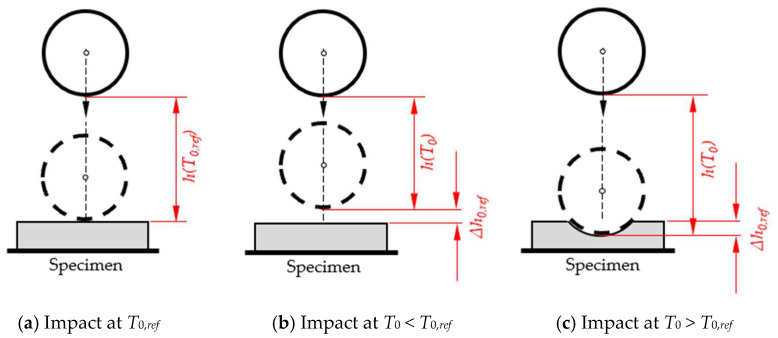
Relation of *T*_0_ to the reference value *T*_0,*ref*_. (**a**) Reference value *T*_0,*ref*_ when impacting on a hard, non-elastic specimen; (**b**) *T*_0_ *< T*_0,*ref*_; (**c**) *T*_0_ *> T*_0,*ref*_ (illustration not to scale).

Reference values *T*_0,*ref*_ serve to identify measurements with the actual height *h* deviating from the prearranged setting. When a soft specimen is tested and *T*_0_ is near to *T*_0*,ref*_ (i.e., the deviation exceeds the standard error, but is smaller than 3 ms), this indicates that either the specimen’s thickness was not 25 mm or that the fine adjustment was inaccurate. Such deviations can result in shorter or longer *T*_0_ (Figure 9). If the measured value T_0_ is less than the reference value *T*_0,*ref*_, then the deviation can only be caused by an incorrect specimen thickness or measurement height. If *T*_0_ is greater than *T*_0,*ref*_, then it is also possible that the deviation is caused by a specimen that is too soft, in which case the acceleration sensors do not correctly detect the impact.

A rapid evaluation of *T*_0_ can be accomplished by using tabulated values for Δ*h* and Δ*T*_0_ = ±1 ms, ±2 ms, ±3 ms. For calculation of the height based on *T*_0_, the free-fall model (Equation (3); [10]) is used for 25 mm height, and the average model (Equation (4); [10]) for 50 mm and 75 mm height. The rationale for choosing the models is given in Section 3.5.1.

(3) $$h=\frac{g\cdot {T_{0}}^{2}}{2},\;\;\;\;\;\;\;\;\;\;\;\frac{∆h}{h}=2\cdot \frac{∆T_{0}}{T_{0}}.\;$$

(4) $$h=-\frac{2\cdot l^{2}}{g\cdot {T_{0}}^{2}}+\frac{l}{g\cdot {T_{0}}^{2}}\cdot \sqrt{4\cdot l^{2}+2\cdot g^{2}\cdot {T_{0}}^{4}},\;∆h=\left|\frac{4\cdot l}{g\cdot {T_{0}}^{2}}-\frac{8\cdot l^{2}+2\cdot g^{2}\cdot {T_{0}}^{4}}{g\cdot {T_{0}}^{2}}\cdot \frac{\sqrt{4\cdot l^{2}+2\cdot g^{2}\cdot {T_{0}}^{4}}}{4\cdot l^{2}+2\cdot g^{2}\cdot {T_{0}}^{4}}\right|\cdot ∆l+\left|\frac{4\cdot l^{2}}{g\cdot {T_{0}}^{3}}-\frac{8\cdot l^{3}}{g\cdot {T_{0}}^{3}}\cdot \frac{\sqrt{4\cdot l^{2}+2\cdot g^{2}\cdot {T_{0}}^{4}}}{4\cdot l^{2}+2\cdot g^{2}\cdot {T_{0}}^{4}}\right|\cdot ∆t.$$

Since Equations (3) and (4) for *h = h*(*T*_0_) are strictly monotonically increasing in the interval [0 s; 1 s], the deviation of *T*_0_ from *T*_0,*ref*_ can be calculated as shown in Equation (5). The monotonicity of *h* as represented in (3) and (4) is examined in detail in File S4.

(5) $$∆h_{0,ref}=\left|h\left(T_{0,ref}\right)-h\left(T_{0}\right)\right|$$

The differences in height Δ*h*_0,*ref*_ for given Δ*t* are tabulated in Table 5. The corresponding calculations are given in File S5.

When a soft specimen is tested and *T*_0_ exceeds *T*_0,*ref*_ for more than 3 ms, we can assume that the accelerometers in the sphere do not report the first impact but a point of time when the sphere already indents the specimen. This will invalidate the calculations made by the STFR.

This explains why the STFR must be constructed with great care, with low tolerances and that the device must be adjusted carefully.

### 3.4. Measurement of the Time and Acceleration in a Soft Specimen

Data generated by these measurements serve to assess the models presented in [10]. The first step was to process the data reported from the accelerometers, by calculating the mean values and standard errors for time and acceleration measurements (Table 6). Raw data and calculations are presented in File S6.

The second step was to analyze the video recordings. The arrangement of the camera was as described in Section 2.3.1.

We used the apparent outline as a mark for the sphere. It was marked when the sphere first came into contact with the specimen. Since the apparent outline of a sphere is generally a conical section (parabola, hyperbola, or ellipse) [13], we had to clarify whether the apparent outline of the sphere in our configuration was approximately a circle and thus our method of placing the center mark on the sphere was justified. This was analyzed using mathematical–geometric methods and is described in detail in File S2. The imaging process in the camera was approximated by a central projection defined by an eye point and an image plane. Further considerations on projection onto a curved image surface can be found in [14].

**Figure 10:**
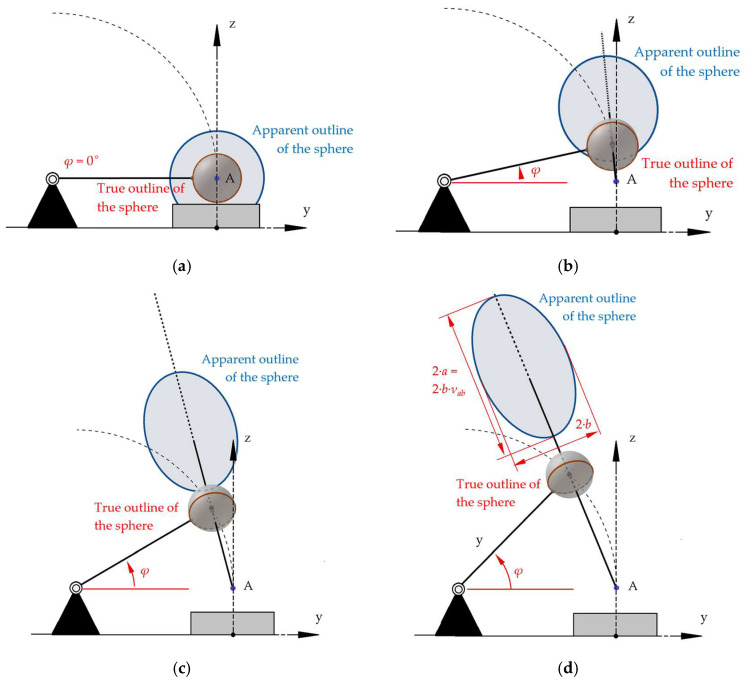
True outline (red) and apparent outline (blue) of the sphere in four positions (i.e., four different elevation angles). The true outline of a sphere is always a circle. (**a**) In the impact position, the apparent outline of the sphere is a circle. (**b**) At a small elevation angle *φ*, the apparent outline is a slightly distorted circle. (**c**,**d**) show that at a higher elevation angle, the apparent outline is clearly elliptical. In (**d**), the length 2·*a* and the width 2·*b* are shown. Figures were constructed with Geogebra Classic ^®^ 5.2.889.0-d, with the radius of the sphere *R* = 15 mm, length of the rod *l* = 100 mm, and the distance between the eye point A and the image plane *n* = 110 mm. The distance *n* does not affect the ratio *v_ab_*, since a change in *n* only causes a parallel shift of the image plane and thus a centric stretching of the image. Details are given in File S7 (illustration not to scale).

The shape and size of the sphere’s apparent outline for different elevation angles *φ* are shown in Figure 10.

The ratio *v_ab_* = *a*:*b* (half the length and half the width of the ellipse) is determined by the elevation angle *φ*. *v_ab_* for angles from 5 to 35° is given in Table 7. For calculations, see File S8.

In order to assess the impact on the STFR, the elevation angles are related to the default heights (Table 8).

Table 8 shows, that for an initial height *h* = 25 mm, the sphere is elevated 8.4565° from the zero position. In this case, the ratio *v_ab_* = 1.0066 at maximum (*v_ab_* for 10° in Table 7); i.e., for 2·*a* = 30 mm, 2·*b* will not exceed 30·1.0066 mm = 30.198 mm. Likewise, 2·*b* will not exceed 30.591 mm and 31.161 mm for *h* = 50 mm and *h* = 75 mm, respectively.

Since the function *v_ab_* = *v_ab_*(*φ*) is strictly monotonic (File S2), these calculated deviations from a circular shape are maximum values that will not be reached in practice.

Data from the image analysis are processed as described in Section 2.3.4. For conversion from pixels to distances in mm, conversion factors are calculated (Table 9).

In the STFR, the accelerometers are activated before the sphere is released. The resulting difference in time differs from measurement to measurement. For comparison of measurements, data rows need to be adjusted by shifting the data along the t- and x-axis.

For each measurement, time *T*_0_, *T*_1_, *d*_0_, and *t_P_*_0_, penetration depth *D* and the maximum height *h_max_* at first rise were determined. Table 10 reports averages and standard errors.

### 3.5. Calculations Using Mathematical Models

We now evaluate previously presented formulae sets [10] that describe the movement of the STFR’s sphere. To this end, we calculate characteristic values from results generated by said formulae and from data reported by the STFR. These characteristic values are compared to data from video image analysis (Section 2.3). This allows us to select the most representative mathematical model.

#### 3.5.1. Calculation of the Initial Height *h* for Time *T*_0_

Validity of measurement is checked by calculating the initial height *h*, based on the time to impact *T*_0_, and by comparing these values with the prearranged height. Calculation of the initial height involves three models ([10]; free fall, Equation (1); hammer model, Equation (3); average model, Equation (6)). Results for the three default conditions are shown in Table 11; calculations are given in File S9.

In the hammer model, the initial height *h* and the maximum error Δ*h* are calculated according to Equation (6).

(6) $$h=-\frac{l^{2}}{g\cdot {T_{0}}^{2}}+\frac{l}{g\cdot {T_{0}}^{2}}\cdot \sqrt{l^{2}+g^{2}\cdot {T_{0}}^{4}},\;∆h=\left|\frac{2\cdot l}{g\cdot {T_{0}}^{2}}-\frac{2\cdot l^{2}+g^{2}\cdot {T_{0}}^{4}}{g\cdot {T_{0}}^{2}}\cdot \frac{\sqrt{l^{2}+g^{2}\cdot {T_{0}}^{4}}}{l^{2}+g^{2}\cdot {T_{0}}^{4}}\right|\cdot ∆l+\left|\frac{2\cdot l^{2}}{g\cdot {T_{0}}^{3}}-\frac{2\cdot l^{3}}{g\cdot {T_{0}}^{3}}\cdot \frac{\sqrt{l^{2}+g^{2}\cdot {T_{0}}^{4}}}{l^{2}+g^{2}\cdot {T_{0}}^{4}}\right|\cdot ∆t.$$

Expectedly, the agreement of calculated *h* to the preadjusted height is best for the free-fall model at low height and for the average model at 50 mm and 75 mm height.

#### 3.5.2. Calculation of the Maximum Penetration Depth *D*

The calculation of the maximum penetration depth *D = h_min_* of the probe in the specimen requires the transition velocity *v_0_*. To this end, the formulae for free fall, Equation (7); hammer model, Equation (8); the average model, Equation (9); and data from Table 6 are used and results displayed in Table 12. The (calculated) transition velocity cannot be directly compared with measured data but is an intermediate result in calculating *D*.

The transition velocity *v_0_* and its maximum error Δ*v_0_* in the free-fall model are calculated with Equation (7) [10].

(7) $$v_{0}=-g\cdot T_{0},\;\;\;\;\;\;\;\;\frac{∆v_{0}}{\left|v_{0}\right|}=\frac{∆T_{0}}{T_{0}}.$$

The transition velocity *v_0_* and its maximum error Δ*v*_0_ in the hammer model are calculated with Equation (8) [10].

(8) $$v_{0}=-\left(1-\frac{h^{2}}{l^{2}}\right)\cdot g\cdot T_{0},\;\;\;\;\;\;\;\;\frac{∆v_{0}}{\left|v_{0}\right|}=\frac{2\cdot h^{2}}{l^{2}-h^{2}}\cdot \left(\frac{∆h}{h}+\frac{∆l}{l}\right)+\frac{∆T_{0}}{T_{0}}.$$

The transition velocity *v*_0_ and its maximum error Δ*v*_0_ in the average model are calculated with Equation (9) [10].

(9) $$v_{0}=-\left(1-\frac{h^{2}}{2\cdot l^{2}}\right)\cdot g\cdot T_{0},\;\;\;\;\;\;\;\;\frac{∆v_{0}}{\left|v_{0}\right|}=\frac{2\cdot h^{2}}{2\cdot l^{2}-h^{2}}\cdot \left(\frac{∆h}{h}+\frac{∆l}{l}\right)+\frac{∆T_{0}}{T_{0}}$$

Equation (10) (taken from [10]) allows calculation of the maximum penetration depth *D* without consideration of the transition velocity *v*_0_. This formula is currently implemented in the STFR.

(10) $$\begin{array}{c} D=g\cdot t_{P0}\cdot \left(T_{0}-\frac{G_{max}\cdot t_{P0}}{6}\right),\\ ∆D=g\cdot \left(\left|T_{0}-\frac{G_{max}\cdot t_{P0}}{3}\right|\cdot ∆t_{P0}+t_{P0}\cdot ∆T_{0}+\frac{{t_{P0}}^{2}}{6}\cdot ∆G_{max}\right). \end{array}$$

Equation (11) is based on a cubic approximation of the curve describing the movement of the sphere in the specimen. The derivation of the formulae is given in [10]. In contrast to Equation (10), *v*_0_ is used.

(11) $$\begin{array}{c} D=\frac{t_{P0}}{6}\cdot \left(2\cdot v_{0}-{g\cdot G}_{max}\cdot t_{P0}\right),\\ ∆D=\frac{\left|v_{0}-{g\cdot G}_{max}\cdot t_{P0}\right|}{3}\cdot ∆t_{P0}+\frac{t_{P0}}{3}\cdot ∆v_{0}+g\cdot \frac{{t_{P0}}^{2}}{6}\cdot ∆G_{max}. \end{array}$$

The maximum penetration depth in the cubic model is calculated using *v*_0_ as derived from the free-fall model, the hammer model, and the average model. Data for *t_P_*_0_ and *G_max_* were taken from Table 5. Data for *v*_0_ were taken from Table 12 and a reference value *D* (calculated in Kinovea) from Table 9. Results are shown in Table 13.

Calculation of the maximum penetration depth *D* with the cubic model yielded the best agreement with the data from the Kinovea analysis (Table 13). For 25 mm height, *v*_0_ is determined by the free-fall model and for 50 mm and 75 mm by the average model.

#### 3.5.3. Calculation of the Maximum Height h_max_ at First Rebound

The maximum height at first rebound, *h_max_*, can be determined by video image analysis and also be calculated in the various models.

The calculation of *h_max_* involves two steps (details see File S12).

- First, the transition velocity *v*_1_, Equation (9), is calculated with the cubic model. This approach requires *D*, which differs between models (see Table 13). Results for *v*_1_ are reported in Table 14.
- Next, the maximum height *h_max_* is calculated by Equation (13) [10], with *D = h_min_* and *v*_1_ from Table 14.

For calculation of *v*_1_, Equation (12), i.e., the cubic model for *v*_1_, is used.

(12) $$v_{1}=-\frac{g\cdot G_{max}\cdot \left(d_{0}-t_{P0}\right)}{2}-\frac{3\cdot D}{d_{0}-t_{P0}},\;∆v_{1}=g\cdot \frac{d_{0}-t_{P0}}{2}\cdot ∆G_{max}+\frac{3}{d_{0}-t_{P0}}\cdot ∆D+\frac{1}{2}\cdot \left|\frac{g\cdot G_{max}\cdot {\left(d_{0}-t_{P0}\right)}^{2}-6\cdot D}{{\left(d_{0}-t_{P0}\right)}^{2}}\right|\cdot \left(∆d_{0}+∆t_{P0}\right).$$

The maximum rebound height is calculated in Equation (13) using *v*_1_ from Table 14.

(13) $$\;\;\;\begin{array}{c} t_{max}=\frac{v_{1}}{g},\;\;\;\;\frac{∆t_{max}}{t_{max}}=\frac{∆v_{1}}{v_{1}},\\ h_{max}=\frac{1}{2}\cdot \frac{{v_{1}}^{2}}{g},\;\;\;\;\frac{∆h_{max}}{h_{max}}=2\cdot \frac{∆v_{1}}{v_{1}}. \end{array}$$

The formulae implemented in the STFR imply that the maximum rebound height is achieved at *T*_1_ [10]. This is based on the assumption that the movement of the sphere in air can be approximated with a quadratic function (‘parabolic trajectory’). We thus examine the validity of Equation (13) by comparing *t_max_* and *h_max_* as calculated by the formula with results reported by the STFR and *h_max, kin_* generated by video image analysis.

Results are presented in Table 15, Table 16 and Table 17; calculations are found in File S13.

Results in Table 15, Table 16 and Table 17 indicate that the calculation of the maximum rebound height *h_max_* should be performed with *v*_1_ calculated by the cubic model, since this gives the best agreement of *h_max_* with *h_max,kin_* from the video image analysis. For 25 mm height, penetration depth *D* is best calculated by the free-fall model, for 50 mm and 75 mm height, the average model is to be preferred.

However, there is a marked difference for 25 mm prearranged initial height between the calculated value for *h* and the corresponding result derived from video image analysis.

#### 3.5.4. Calculation of Energy Restitution *E_R_*

Energy restitution *E_R_* is an important parameter, since can be used (in a normalized form) to compare different initial heights. In order to determine the best-fit mathematical model to be implemented in future versions of the STFR, we compare the currently implemented free-fall model, Equation (14), with a model based on *h_max_* at first rebound, Equation (15), and a model including *v*_1_ (speed when the sphere returns from the specimen; [10]), Equation (16). Results are presented in Table 18.

The mode of calculation of Energy Recovery *E_R_* as currently implemented in the STFR is shown in Equation (14).

(14) $$\begin{array}{cc} E_{R}={\left(\frac{T_{1}}{T_{0}}\right)}^{2}, & \frac{∆E_{r}}{E_{r}}=2\cdot \left(\frac{∆T_{0}}{T_{0}}+\frac{∆T_{1}}{T_{1}}\right). \end{array}$$

The mode of calculation of Energy Recovery *E_R_* considering the initial height and the maximum height at first rebound is shown in Equation (15). This formula is particularly useful for calculation of *E_R_* from the video image analysis data.

(15) $$\begin{array}{cc} E_{R}=\frac{h_{max}}{h}, & \frac{∆E_{r}}{E_{r}}=\frac{∆h}{h}+\frac{∆h_{max}}{h_{max}}. \end{array}$$

The mode of calculation of Energy Recovery *E_R_* considering the calculated transition velocity *v*_1_ is shown in Equation (16).

(16) $$\begin{array}{cc} E_{R}=\frac{{v_{1}}^{2}}{2\cdot g\cdot h}, & \frac{∆E_{r}}{E_{r}}=\frac{∆h}{h}+2\cdot \frac{∆v_{1}}{v_{1}} \end{array}.$$

Obviously, calculation of *E_R_* gives best results for low initial height (25 mm) with the formula already implemented in the STFR, Equation (14), whereas at 50 mm and 75 mm initial height, the cubic model, Equation (16), gives the best agreement.

#### 3.5.5. Calculation of the Spring Constant near to the Specimen Surface and of the Damping Constant near to the Specimen Surface

Other than the parameters calculated in Section 3.5.1, Section 3.5.2, Section 3.5.3 and Section 3.5.4, the spring constant near the specimen surface and the damping constant near the specimen surface cannot be compared with results from the STFR measurements and video image analysis.

The spring constant near the specimen surface [10] can be calculated with Formula (17) or, based on a power series calculation, with Formula (18) [10].

(17) $$\begin{array}{cc} k=\frac{{G_{max}}^{2}\cdot m}{{T_{0}}^{2}}, & \;\;\;\;\;\;\;\;\frac{∆k}{k}=2\cdot \left(\frac{∆G_{max}}{G_{max}}+\frac{∆T_{0}}{T_{0}}\right)+\frac{∆m}{m} \end{array}.$$

(18) $$k_{h}=\left(\frac{g^{2}\cdot G_{max}\cdot \left(G_{max}-1\right)}{{v_{0}}^{2}}+\frac{2\cdot g\cdot \left(G_{max}-1\right)}{t_{P0}\cdot v_{0}}+\frac{2}{{t_{P0}}^{2}}-\frac{{v_{0}}^{2}}{l^{2}}\right)\cdot m,\;\begin{array}{c} \;\;\;\;\;\;\;\;\;\;\;\;\;\;\;\;\;\;\;\;\;\;\;\;\;\;\;\;\;\;\;\;\;\;\;\;\;\;\;\;\;\;\;\;∆k_{h}=\left|\frac{g^{2}\cdot G_{max}\cdot \left(G_{max}-1\right)}{{v_{0}}^{2}}+\frac{2\cdot g\cdot \left(G_{max}-1\right)}{t_{P0}\cdot v_{0}}+\frac{2}{{t_{P0}}^{2}}-\frac{{v_{0}}^{2}}{l^{2}}\right|\cdot ∆m+m\cdot g\\ \;\;\;\;\;\;\;\;\;\;\;\;\;\;\;\;\;\;\;\;\;\;\;\;\;\;\;\;\;\;\;\;\;\;\;\;\;\;\;\;\;\;\;\;\;\;\;\;\;\;\;\;\;\;\;\;\;\;\;\;\;\;\;\;\;\;\;\;\;\;\;\;\;\;\;\;\;\;\;\;\;\;\;\;\;\cdot \left|g\cdot \frac{2\cdot G_{max}-1}{{v_{0}}^{2}}+\frac{2}{t_{P0}\cdot v_{0}}\right|\cdot ∆G_{max}+\frac{2\cdot m}{{t_{P0}}^{2}}\cdot \left|\frac{G_{max}-1}{v_{0}}\cdot g+\frac{2}{t_{P0}}\right|\\ \;\;\;\;\;\;\;\;\;\;\;\;\;\;\;\;\;\;\;\;\;\;\;\;\;\;\;\;\;\;\;\;\;\;\;\;\;\;\;\;\;\;\;\;\;\;\;\;\;\;\;\;\;\;\;\;\;\;\;\;\;\;\;\;\;\;\;\;\;\;\;\;\;\;\;\;\;\;\;\;\;\;\cdot ∆t_{P0}+2\cdot m\cdot \left|\frac{{g^{2}\cdot G}_{max}\cdot \left(G_{max}-1\right)}{{v_{0}}^{3}}+g\cdot \frac{G_{max}-1}{t_{P0}\cdot {v_{0}}^{2}}+\frac{v_{0}}{l^{2}}\right|\cdot ∆v_{0}\\ +\frac{2\cdot {v_{0}}^{2}\cdot m}{l^{3}}\cdot ∆l. \end{array}$$

Assuming that the specimen behaves viscoelastically near the surface (Kelvin body), we employed power series calculations to elaborate two sets for calculation of the damping constant *c* ([10]). Whereas Formula (19) uses a quadratic approximation, Formula (20) is based on a cubic approximation for the equation of motion. Since both formulae use Taylor polynomials, we can assume that Formula (20) is more appropriate for deeper penetration of the probe into the specimen.

The damping constant *c_s_* near the specimen surface is given by Equation (19).

(19) $$c_{s}=-g\cdot \frac{G_{max}+1}{v_{0}}\cdot m,\;∆c_{s}=g\cdot \left(\left|\frac{m}{v_{0}}\right|\cdot ∆G_{max}+\frac{G_{max}+1}{{v_{0}}^{2}}\cdot m\cdot ∆v_{0}+\left\lceil \frac{G_{max}+1}{v_{0}}\right\rceil \cdot ∆m\right).$$

The damping constant *c_h_* for deeper penetration is given by Equation (20).

(20) $$c_{h}=\left(g\cdot \frac{G_{max}-1}{v_{0}}+\frac{2}{t_{P0}}\right)\cdot m,\;∆c_{h}=\left|g\cdot \frac{G_{max}-1}{v_{0}}+\frac{2}{t_{P0}}\right|\cdot ∆m+\frac{g\cdot m}{\left|v_{0}\right|}\cdot ∆G_{max}+\frac{2\cdot m}{{t_{P0}}^{2}}\cdot ∆t_{P0}+\frac{g\cdot m\cdot \left(G_{max}-1\right)}{{v_{0}}^{2}}\cdot ∆v_{0}.$$

Results generated from Formulae (19) and (20) are displayed in Table 19.

**Figure 11:**
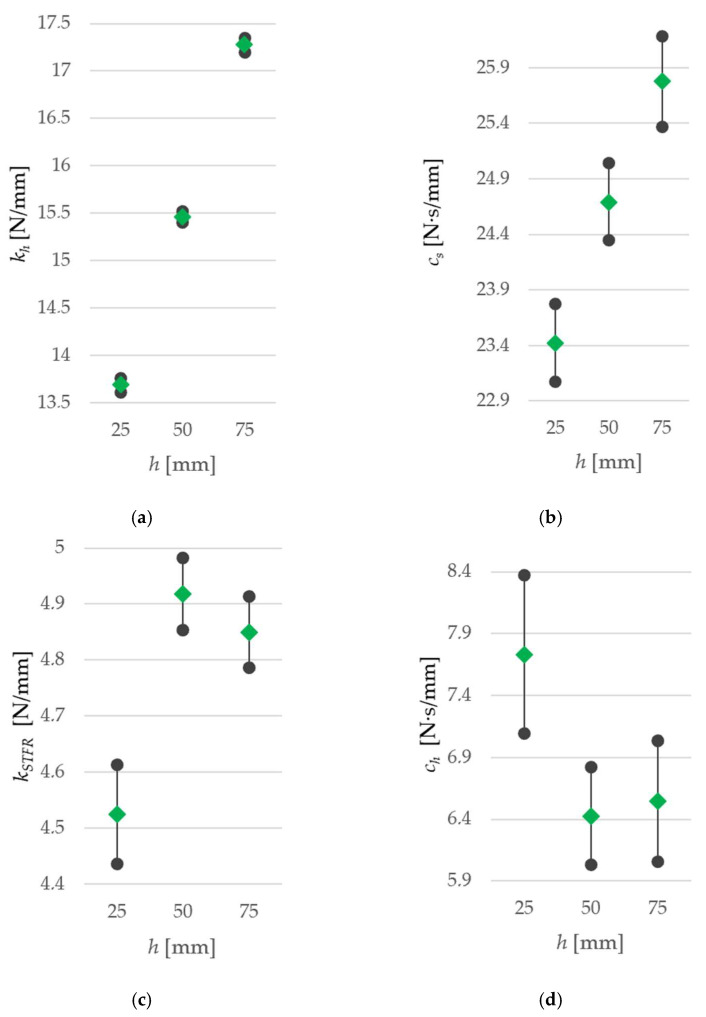
Average-Max-Min chart: data range for (**a**) spring constant *k_h_*, (**b**) damping constant *c_s_*, (**c**) spring constant *k_STFR_*, and (**d**) damping constant *c_h_*. Calculations are given in File S16.

Values for the spring constant *k_h_* and for the damping constant *c_s_* increase with increasing initial height *h* (Figure 11a,b). This trend cannot be identified for *k_STFR_* (Figure 11c) and *c_h_* (Figure 11d) since the ranges of results at *h* = 50 mm and *h* = 75 mm overlap.

Explanations of studied theoretical material parameters are given below.

- The spring constant *k_STFR_* considers the shape of the sphere and is valid in the near-to-surface section of the specimen (File S17).
- The characteristic values *k_h_*, *c_s_* and *c_h_* do not consider the shape of the sphere (i.e., the sphere is represented as a point, the trajectory is approximated by power series calculations; [8]) and will be valid also for large distances from the specimen surface.
- None of the abovementioned characteristic values can consider the actual penetration, displacement, or deformation processes in the specimen. Likewise, the non-linear characteristic of the damping due to the shape of the sphere, with the contact area increasing with increasing penetration, is not considered. Finally, adhesiveness of the specimen’s surface is neglected.

### 3.6. Adaptation of the Formula Set for Large Heights (50 mm and 75 mm)

The findings presented in Section 3.5 suggest using different sets of formulae for the VRT and the STFR. Recommended sets of formulae are listed in Table 20, Table 21, Table 22, Table 23, Table 24, Table 25 and Table 26.

Table 20 compares formulas for calculating the pre-arranged initial height *h*. It is recommended that a new set of formulas be used.

Table 21 compares formulas for calculating the penetration depth *D*. Since the transition velocity *v*_0_ is also required for the calculation, a recommendation is also made for this formula. The use of the new set of formulas is recommended for calculating the penetration depth in the STFR software.

Table 22 shows the formula for calculating Young’s Modulus E*. In contrast to the original formula, the absolute value of the penetration depth *D* is used.

Table 23 shows the formulas for calculating the spring constant. Both the original formula for *K = k_STFR_* and a modified formula for *k_h_* are recommended.

Table 24 shows the new formulas for calculating damping constants *c_s_* and *c_h_*.

Table 25 lists the formulas for calculating Energy Recovery *E_R_*. In the modified formula collection, the original equations are used for *h* = 25 mm ± 1 mm, while newly generated formulas are used for heights *h* of 50 mm ± 1 mm and 75 mm ± 1 mm. The exit velocity *v*_1_ must also be calculated for the new formulas.

Table 26 shows the original formulae for calculating the resonance frequency f_n_. These formulae remain unchanged.

### 3.7. A MS-Access^®^-Based Application for Data Processing

**Figure 12:**
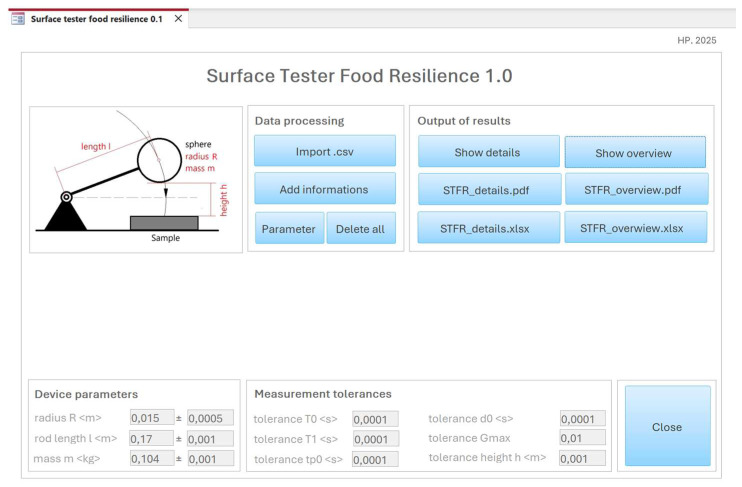
User interface of the data processing software Surface Tester Food Resilience 1.0. (Note: Since a german MS Access version was used, the decimal separator is a comma and not a dot).

For rapid and convenient data handling and processing, an MS Access-based application was created. The user interface is shown in Figure 12.

This application processes data generated by the STFR. It is used to validate formulae and to calculate characteristic values using different models (Section 2.4). The transition velocity *v_0_* is calculated by both the free fall and the average model.

Functions and operation of the application are as follows:

- Function “Import.csv”:
  ○ Loads data from the file SURF_TST.csv and creates a directory /data. Imported data are stored in a backup file, SURF_TST_1_backup.xlsx in the directory /data.
  ○ Groups results into data series, according to date and initial height.
  ○ Performs calculations.

- Function “Add Information”:
  ○ Allows entry of data on analysis and on specimen details; allows deletion of data series.

- Function “Parameter”:
  ○ Allows us to define tolerances. Changes do not automatically trigger a recalculation of data.

- Function “Delete all”:
  ○ Clears all tables and allows to reduce the size of the access file.

- Functions “Show details” and “Show overview”:
  ○ Display reports on the screen.

- Functions “STFR_details.pdf” and “STFR_overview.pdf”:
  ○ Save the reports in *.pdf format in the directory/data.

- Functions “STFR_details.xlsx” and “STFR_overview.xlsx”:
  ○ Save the data in *.xlsx format in the directory/data.

**Figure 13:**
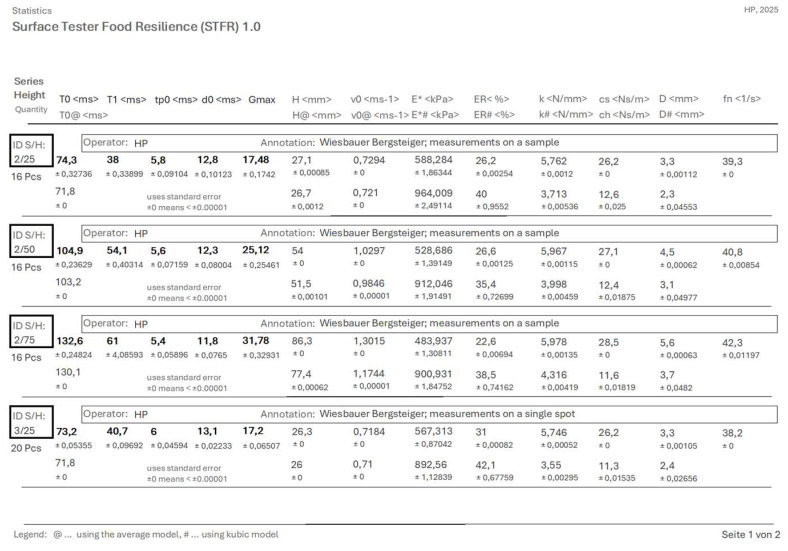
Screenshot of the overview protocol page. This page gives average results of specimens, standard error and maximum error. Measured data are in bold, calculated values in normal letters. (Note: Since a german MS Access version was used, the decimal separator is a comma and not a dot).

The software allows us to present replicates of each individual specimen, together with descriptive statistics or an overview, displaying mean ± standard error per series of measures (i.e., specimens). Characteristic values and their maximum errors are calculated by both the average and the cubic model. Figure 13 shows a screenshot of the overview protocol page.

## 4. Discussion

### 4.1. Benefits of High-Frequency Video Analysis for Movement Analysis

The used high-frequency video is state of the art in medicine and sports [15,16] and also in educational settings [17] and allows us to study fast moving objects. This motivated us to employ video analysis to retrieve data for the validation of our newly developed formulae ([8,10]). While recording frequencies of 50 Hz are sufficient for sports motor movement analyses [18], a significantly higher resolution is required due to the low range of motion of a maximum fall height of 75 mm. The use of a special high-speed camera proved necessary, since smartphone-based systems [19,20] could not generate the required number of frames per unit time.

### 4.2. Specific Issues of High-Frequency Video Analysis for Analysis of the Movement of the STFR Sphere

In sport sciences, the dimensions of the objects under study are usually favorable for setting marking points, although markerless systems have been proven effective in motion analysis [21]. Setting marks on the sphere of the STFR proved to be difficult due to the sphere’s small dimensions. Because the images of the marks appeared blurred in the video frames, the apparent outline of the sphere was used for video analysis. We found that the apparent outline of a sphere maintains approximately circular at all heights (up to 75 mm) due to the distance between the camera (focus) and the sphere. Based on the identification of the apparent outline, we used its center as measuring mark. The accuracy of the image analysis was best at the point of impact and was slightly lower at the point of release. The accuracy of results is not only affected by positioning the optical path of the camera but also by dimension and resolution of the CMOS sensor of the camera and spherical aberrations of the lens unit. Some of these issues could be overcome by the use of more cameras (giving two-dimensional data) from different angles and appropriate software for processing multiple two-dimensional records into a three-dimensional data set [22]. Such equipment, however, was not available.

### 4.3. The Impact of the Initial Position of the Probe on the Measurements and Results

The initial position of the sphere has a major effect on the results. Notably, this refers not only to the initial height (vertical line from the lowest point of the sphere to surface of the specimen) but also to the elevation angle. The mathematical description of the geometry was compared with practical experiments. In our setting, the guiding rod was in horizontal position at the time point of the first contact of the sphere to the specimen’s surface. This adjustment should be maintained in further use of the STFR. This also requires a defined specimen thickness or a height-adjustable receptacle for the specimen. 

### 4.4. Benefits and Limitations of an Extension of the Measurement Range of the STFR

The formulae currently implemented in the STFR were based on a free-fall design, which is an acceptable approximation of low elevation angles, corresponding to an initial height of 17 mm [8]. A new set of formulae considering the hammerhead-like movement of the sphere for initial height up to 75 mm has been presented [10]. We studied initial heights of 25 mm, 50 mm, and 75 mm and identified the formulae describing the movement and derived characteristic values more accurately. The benefit of different initial height is that, due to the different magnitude of momentum at impact, the specimen characteristics can be assessed not only near the surface but also, due to increased penetration depth, more in the interior of the specimen. Admittedly, the use of Taylor polynomials [10] implies that the formulae become less accurate the deeper the penetration depth.

Further studies will explore if testing at different penetration depths (i.e., different initial height) could give information on the material characteristics on non-homogenous specimens with a layered structure, e.g., bananas with the peel on the surface and the interior pulp or fried meat products with different characteristics of the crust and the interior part. Current approaches to study such specimens rely on modifications of Texture Profile Analysis with complex data processing procedures [23].

### 4.5. Improvements for the STFR Formula Apparatus and Remaining Inherent Limitations

We used high-frequency video recordings to identify which of the mathematical-physical formulae presented in Section 3.5 are most appropriate for specimen analysis.

The formulae for initial height, maximum penetration depth, and Energy Recovery could be related to results from the video analysis. Formulae for spring constant and for damping constant could not be compared with the results from actual measurements.

Since the STFR was designed for homogenous viscoelastic bodies, specimens with markedly different characteristic are not suitable for testing, e.g., bananas. If testing of such specimens is desired, modifications in the formulae are required. Likewise, sticky surfaces might affect the rebound phase. Whereas such characteristics are taken into account in Texture Profile Analysis (“adhesiveness”; [24]), this condition is not yet implemented in the STFR. Such limitations are not unusual in various texture testing methodologies. Likewise, the modeling we applied is assuming a horizontal plane. This was motivated by the fact that the device was primarily intended for testing meat products and cheese, which can be sliced/trimmed to defined dimensions. For testing samples with inclined surfaces, the formulae need to be adjusted. Uneven surfaces could result in differences in damping.

As regards the need for standardized dimensions of the specimens, this is common for shear force (e.g., a 12.7 mm diameter cylindrical core, or a prism with 1 cm × 1 cm square cross-section) [2,3,6,7,9] and also for compression tests and TPA; the height must at least be known, and it is better, for the sake of reproducibility, if adjusted to a defined size. Basically, the findings in our paper give evidence that the top surface of the specimen must be adjusted in a way that, when the sphere is at rest at that surface, the guiding rod is in vertical position. This could be accomplished by adjusting the height of the stand carrying the swivel for the guiding rod. The rig we presented in the manuscript does not foresee such a height adjustment, since we wanted a rugged stand for making high-speed videos.

Finally, the physical models we used (free fall, hammer model, and average model) are simplifications of a contact phenomenon which is more complex in reality. Thus, a comprehensive model would need to include multibody impact, viscoelastic and viscoplastic impact, and the effects of local compliance and friction [25,26]. Still, the formulae elaborated in this manuscript and in [10] represent an important improvement in the understanding and mathematical processing of the STFR data.

### 4.6. Perspectives for the Use of the STFR with Improved Formula Apparatus

Out of a previously elaborated set of formulae [10], we could identify formulae for accurate calculation of specimen surface characteristics and present an application integrating these formulae in the test procedure. The application of this improved testing device in real foods and comparison with results from established texture analyzers will be studied in future projects.

## 5. Final Conclusions

- For an impact-based accelerometer-equipped surface tester designed for solid foods with viscoelastic characteristics, formulae describing the arc-shaped path of the probe and derived material characteristic values were validated by analysis of high-speed video recordings.
- We elaborated reference time values for the first falling phase of the sphere, which can be used to check the validity of measured data, and give an indication on sources of error (e.g., unadjusted thickness of the specimen).
- We present formulae for penetration depth and Energy Recovery, based on a free-fall model for 25 mm drop height, and on an average model (hammer and free fall) for drop heights of 50 mm and 75 mm.
- These formulae are the basis for further studies relating the STFR-generated results for spring constant and penetration depth to TPA-generated values for hardness and for Energy Recovery (STFR) with Resilience (TPA), obtained from real food samples. For a comparison of TPA variables relying on two compression–relaxation cycles, the formulae presented in our manuscript will be simply applied on the first and on the second drop–bounce cycle.
- Limitations of this method are the need for horizontal, plane specimen surfaces. Inhomogeneous or multi-layered specimens have not been considered and some simplifications on impact mechanics have been made.

## Figures and Tables

**Table 1:** Measurements carried out by the STFR and units as reported by the STFR (taken from [10]).

Variable	Meaning	Physical Unit
*T*_0_	Duration from release of the sphere to the first impact (=first free-flight/-fall phase)	Seconds, [s]
*d*_0_	Duration of the first contact phase of the sphere with the specimen surface	Milliseconds, [ms]
*t_P_*_0_	Duration from first sphere-specimen contact to the extremum of acceleration	Milliseconds, [ms]
2*·T*_1_	Duration of the second free-flight phase	Seconds, [s]
*G_max_*	Factor for calculating the peak acceleration	Gravitational acceleration, [g-unit]

The value *G_max_* is a factor with which the acceleration *a_max_ = G_max_·g* can be calculated. For example, a measured value of *G_max_* = 10 corresponds to a peak acceleration of *a_max_* = 10·9.81 m·s^−2^ = 98.1 m·s^−2^.

**Table 2:** Mean values and standard errors of time to impact *T*_0_ (*n* = 5). Settings as in Figure 4.

	Specimen Thickness, [mm]		
	25	50	75
*x_offset_*, [mm]	0	25	50
Measured *T*_0_, [ms]	71.979 ± 0.008	74.521 ± 0.047	78.375 ± 0.023
Relative deviation of *T*_0_ in relation to the standard specimen thickness (25 mm)	0%	3.53%	8.89%

Prearranged height *h* = (25 ± 1) mm.

**Table 3:** Relative deviation *rd_φ_* and its maximum error of the angle *φ*_1_ in the “high position” compared to *φ*_0_ in the standard position, calculated according to Equation (1). This value applies also when the corresponding arcs are compared (*rd_b_*), Equation (2). Calculation of the data presented in this Table is given in File S3.

x_offset_ [mm]	Relative Deviation rd_φ_ and rd_b_, for Initial Height h=		
	25 mm ± 1 mm	50 mm ± 1 mm	75 mm ± 1 mm
0	0.00% ± 0.04%	0.00% ± 0.09%	0.00% ± 0.15%
5	0.26% ± 0.07%	0.51% ± 0.12%	0.76% ± 0.17%
10	0.62% ± 0.09%	1.11% ± 0.14%	1.71% ± 0.20%
15	1.07% ± 0.12%	1.83% ± 0.16%	2.77% ± 0.23%
20	1.62% ± 0.14%	2.66% ± 0.19%	3.97% ± 0.26%
25	2.27% ± 1.16%	3.61% ± 0.22%	5.33% ± 0.29%
30	3.02% ± 0.19%	4.70% ± 0.24%	6.88% ± 0.33%
35	3.90% ± 0.21%	5.93% ± 0.27%	8.63% ± 0.37%
40	4.90% ± 0.24%	7.32% ± 0.30%	10.62% ± 0.41%
45	6.03% ± 0.26%	8.89% ± 0.34%	12.88% ± 0.47%
50	7.31% ± 0.29%	10.66% ± 0.37%	15.47% ± 0.53

For a given length of the rod *l* = 170 mm ± 1 mm, an elevation of the specimen *x_offset_* = 50 mm ± 1 mm and an initial sphere-to-specimen distance of 25 mm ± 1 mm, the angle corresponding to the travel of the sphere is 7.31% ± 0.29% greater than for *x_offset_* = 0 mm ± 1 mm.

**Table 4:** Reference values *T*_0,*ref*_ (mean ± standard error; *n* = 5).

Initial Height *h* [mm]	*T*_0,*ref*_ [ms]
25 ± 1	71.979 ± 0.008
50 ± 1	103.648 ± 0.006
75 ± 1	131.298 ± 0.013

*h* = initial height.

**Table 5:** Deviations in initial height *h*, depending on the deviations of the time from release to impact to the reference time.

*h* [mm]	Δ*h*_0,*ref*_ [mm] for Selected Δ*t* [ms]					
	Δ*t* = 1 ms		Δ*t* = 2 ms		Δ*t* = 3 ms	
	*T*_0_ < *T*_0,*ref*_	*T*_0_ > *T*_0,*ref*_	*T*_0_ < *T*_0,*ref*_	*T*_0_ > *T*_0,*ref*_	*T*_0_ < *T*_0,*ref*_	*T*_0_ > *T*_0,*ref*_
25 ± 1	0.701	0.711	1.393	1.432	2.074	2.162
50 ± 1	0.888	0.893	1.772	1.789	2.651	2.690
75 ± 1	0.948	0.948	1.896	1.895	2.844	2.842

How to use this table: Assuming that a time of *T*_0_ = 73.8 ms was measured at a height of (25 ± 1) mm, the corresponding reference time is *T*_0,*ref*_ = (71.979 ± 0.008) ms (as specified in Table 4). Because *T*_0_ > *T*_0,*ref*_ and 1 ms < Δ*t* = 73.8 ms − 71.979 ms = 1.821 ms < 2 ms, the deviation in height is in the range of 0.711 mm to 1.432 mm. Given a correct height adjustment, we conclude that the thickness of the specimen deviates from 25 mm for more than 0.711 mm but less than 1.432 mm.

**Table 6:** Descriptive statistics (*n* = 5; average ± standard error) for the data reported by the STFR.

Initial Height *h* [mm]	*T*_0_ [ms]	*T*_1_ [ms]	*t_P_*_0_ [ms]	*d*_0_ [ms]	*G_max_* [g-Units]
25 ± 1	71.151 ± 0.052	45.645 ± 0.047	7.377 ± 0.116	16.331 ± 0.009	14.842 ± 0.063
50 ± 1	102.466 ± 0.037	62.56 ± 0.012	7.174 ± 0.057	15.402 ± 0.005	22.282 ± 0.031
75 ± 1	129.683 ± 0.105	76.224 ± 0.125	6.81 ± 0.062	14.534 ± 0.023	28.004 ± 0.027

**Table 7:** *v_ab_* for different elevation angles *φ*.

*φ* [deg]	*v_ab_*
0	1
5	1.0025
10	1.0066
15	1.0124
20	1.0197
25	1.0285
30	1.0387
35	1.0502

**Table 8:** Maximum elevation angle *φ* of the sphere at the default heights *h*, see File S2.

*h* [mm]	*φ* [deg]
25	8.4565
50	17.1045
75	26.1790

**Table 9:** Conversion factors (mean ± standard error) for calculating mm from height in pixels; 1 mm corresponds to approx. 7 (6.6–6.8) pixels. The calculations are given in File S6.

Initial Height *h* [mm]	Conversion Factors *cf* from Pixel [px] to [mm]
25 ± 1	0.151 ± 0.000
50 ± 1	0.148 ± 0.000
75 ± 1	0.148 ± 0.000

Conversion factors are calculated from 5 replicate measurements. The mathematical formula is described in Section 2.3.4.

**Table 10:** Characteristic values for the movement of the sphere, taken from video image analysis (mean ± standard error). Calculations are given in File S9.

Initial Height *h* [mm]	*T*_0_ [ms]	*T*_1_ [ms]	*t_P_*_0_ [ms]	*d*_0_ [ms]	*D* [mm]	*h_max_* [mm]
25 ± 1	71.290 ± 0.462	45.258 ± 0.113	7.71 ± 0.159	16.36 ± 0.223	3.135 ± 0.058	10.547 ± 0.032
50 ± 1	102.405 ± 0.240	63.13 ± 0.082	7.095 ± 0.059	15.535 ± 0.123	4.172 ± 0.049	19.794 ± 0.043
75 ± 1	129.475 ± 0.160	76.023 ± 0.426	7.095 ± 0.273	14.878 ± 0.139	4.658 ± 0.083	28.981 ± 0.109

**Table 11:** Calculation of the initial height based on the measured *T*_0_ (average ± maximum error).

Prearranged Initial Height [mm]	Measured *T*_0_ [ms]	Computed Initial Height *h* [mm] Using Different Models		
		Free-Fall Model	Hammer Model	Average Model
		Formula (1)	Formula (2)	Formula (3)
25 ± 1	71.151 ± 0.052	24.832 ± 0.036	24.323 ± 0.04	24.572 ± 0.038
50 ± 1	102.466 ± 0.037	51.499 ± 0.037	47.482 ± 0.07	49.331 ± 0.056
75 ± 1	129.683 ± 0.105	82.491 ± 0.134	68.29 ± 0.195	74.558 ± 0.177

**Table 12:** Transition velocity *v*_0_ computed from the measured time to first impact *T*_0_ in various models; calculations in File S10.

Prearranged Initial Height [mm]	Measured *T*_0_ [ms]	Computed Velocity *v*_0_ [m/s]		
		Free-Fall Model	Hammer Model	Average Model
		Formula (7)	Formula (8)	Formula (9)
25 ± 1	71.151 ± 0.052	−0.698 ± 0.001	−0.683 ± 0.002	−0.69 ± 0.001
50 ± 1	102.466 ± 0.037	−1.005 ± 0.000	−0.918 ± 0.005	−0.962 ± 0.003
75 ± 1	129.683 ± 0.105	−1.272 ± 0.001	−1.025 ± 0.01	−1.148 ± 0.006

**Table 13:** Comparison of the maximum penetration depth *D* based on measured *T*_0_, *t_P_*_0_, the velocity *v*_0_, and the acceleration *G_max_*. Calculations are shown in File S11.

				$\mathrm{Maximum}\;\mathrm{Depth}\;\left|D\right|$ [mm]				
	Values Reported from STFR			Measured	Calculated	Cubic Model; *v*_0_ Computed Using …		
Prearranged Initial Height [mm]	*T*_0_ [ms]	*t_P_*_0_ [ms]	*G_max_* [g-Units]	Kinovea	STFR	Free-Fall Model	Hammer Model	Average Model
25 ± 1	71.151 ± 0.052	7.377 ± 0.116	14.842 ± 0.063	3.135 ± 0.058	3.828 ± 0.049	3.037 ± 0.075	3.000 ± 0.079	3.017 ± 0.076
50 ± 1	102.466 ± 0.037	7.173 ± 0.057	22.282 ± 0.031	4.172 ± 0.049	5.333 ± 0.033	4.278 ± 0.052	4.070 ± 0.062	4.175 ± 0.058
75 ± 1	129.683 ± 0.105	6.810 ± 0.062	28.004 ± 0.027	4.658 ± 0.083	6.540 ± 0.049	5.010 ± 0.069	4.445 ± 0.084	4.729 ± 0.078
Used formulae					(10)	(7), (11)	(8), (11)	(9), (11)

**Table 14:** Transition velocity *v_1_* based on measured data for *t_P_*_0_, *d*_0_, the maximum penetration depth *D,* and the acceleration *G_max_*. Calculations are given in File S12.

				Calculation of *v*_1_ [m/s] Using Maximum Penetration Depth *D* [mm]			
	Measured by STFR			Calculated	Calculation of *D* [mm] in the Cubic Model (Formula (11)) Using *v*_0_ [m/s] in …		
Prearranged Initial Height [mm]	*t_P_*_0_ [ms]	*d*_0_ [ms]	*G_max_* [g-Units]	STFR	Free-Fall Model	Hammer Model	Average Model
25 ± 1	7.377 ± 0.116	16.331 ± 0.009	14.842 ± 0.063	0.634 ± 0.023	0.366 ± 0.051	0.353 ± 0.052	0.359 ± 0.052
50 ± 1	7.174 ± 0.057	15.402 ± 0.005	22.282 ± 0.031	1.045 ± 0.023	0.661± 0.039	0.585 ± 0.042	0.623 ± 0.040
75 ± 1	6.810 ± 0.062	14.534 ± 0.023	28.004 ± 0.027	1.479± 0.041	0.885 ± 0.061	0.665 ± 0.065	0.775 ± 0.063
Used formulae				(10), (12)	(7), (11), (12)	(8), (11), (12)	(9), (11), (12)

**Table 15:** Calculated values for the maximum height *h_max_* at first rebound, Equation (13) and of *t_max_*, using the transition velocity values generated by different models (Table 14) with measured *T*_1_ and the corresponding value for *h_max,kin_* derived from video image analysis. Height *h* = 25 mm ± 1 mm.

Reported by STFR	Calculated				Determined by Video Analysis
*T*_1_ [ms]	*v*_1_ [m/s]	Remark: *v*_1_ Uses …	*h_max_* [mm]	*t_max_* [ms]	*h_max,kin_* (Kinovea) [mm]
45.645 ± 0.047	0.634 ± 0.030	maximum depth *D*, reported from STFR	20.485 ± 1.934	64.625 ± 3.050	10.547 ± 0.031
	0.366 ± 0.051	maximum depth *D* (free fall) from the cubic model	6.813 ± 1.917	37.269 ± 5.244	
	0.353 ± 0.052	maximum depth *D* (hammer) from the cubic model	6.359 ± 1.883	36.005 ± 5.331	
	0.359 ± 0.052	maximum depth *D* (average) from the cubic model	6.565 ± 1.889	36.586 ± 5.264	
Used formulae			(13)	(13)	

**Table 16:** Calculated values for the maximum height *h_max_* at first rebound, Equation (13), and of *t_max_*, using the transition velocity values generated by different models (Table 14) with measured *T*_1_ and the corresponding value for *h_max,kin_* derived from video image analysis. Height *h* = 50 mm ± 1 mm.

Reported by STFR	Calculated				Determined by Video Analysis
*T*_1_ [ms]	*v*_1_ [m/s]	Remark: *v*_1_ Uses …	*h_max_* [mm]	*t_max_* [ms]	*h_max, kin_* (Kinovea) [mm]
62.560 ± 0.012	1.045 ± 0.023	maximum depth *D*, reported from STFR	55.664 ± 2.405	106.529 ± 2.301	19.794 ± 0.043
	0.661± 0.039	maximum depth *D* (free fall) from the cubic model	22.238 ± 2.605	67.332 ± 3.944	
	0.585 ± 0.042	maximum depth *D* (hammer) from the cubic model	17.424 ± 2.477	59.602 ± 4.237	
	0.623 ± 0.040	maximum depth *D* (average) from the cubic model	19.781 ± 2.566	63.504 ± 4.119	
Used formulae			(13)	(13)	

**Table 17:** Calculated values for the maximum height *h_max_* at first rebound, Equation (13), and of *t_max_*, using the transition velocity values generated by different models (Table 14) with measured *T*_1_ and the corresponding value for *h_max,kin_* derived from video image analysis. Height *h* = 75 mm ± 1 mm.

Reported by STFR	Calculated				Determined by Video Analysis
*T*_1_ [ms]	*v*_1_ [m/s]	Remark: *v*_1_ Uses …	*h_max_* [mm]	*t_max_* [ms]	*h_max,kin_* (Kinovea) [mm]
76.224 ± 0.125	1.479 ± 0.041	maximum depth *D*, reported from STFR	111.460 ± 6.127	150.744 ± 4.143	28.981 ± 0.109
	0.885 ± 0.061	maximum depth *D* (free fall) from the cubic model	39.884 ± 5.498	90.174 ± 6.215	
	0.665 ± 0.064	maximum depth *D* (hammer) from the cubic model	22.552 ± 4.373	67.807 ± 6.574	
	0.775 ± 0.063	maximum depth *D* (average) from the cubic model	30.651 ± 4.994	79.050 ± 6.440	
Used formulae			(13)	(13)	

**Table 18:** Calculation of Energy Recovery *E_R_* (mean ± standard error).

Prearranged Initial Height [mm]	KINOVEA *E_R_* Based on *h_max_*	STFR *E_R_*	*E_R_* Based on STFR: *v*_1_	*E_R_* Based on Cubic Model: *v*_1_ (Free Fall)	*E_R_* Based on Cubic Model: *v*_1_ (Hammer)	*E_R_* Based on Cubic Model: *v*_1_ (Average)
25 ± 1	42.290% ± 4.296%	41.155% ± 0.354%	81.940% ± 11.013%	27.252% ± 8.758%	25.435% ± 8.549%	26.262% ± 8.608%
50 ± 1	39.588% ± 2.217%	37.276% ± 0.111%	111.328% ± 7.036%	44.475% ± 6.100%	34.849% ± 5.652%	39.562% ± 5.924%
75 ± 1	38.641% ± 1.708%	34.547% ± 0.491%	148.613% ± 10.151%	53.179% ± 8.039%	30.069% ± 6.231%	40.867% ± 7.204%
Used formulae	(15)	(14)	(16)			

Height *h* was the prearranged initial height; values for *h_max_* were taken from Table 10, those for *T*_0_ from Table 6, and those for *v*_1_ from Table 14; see also File S14. A combination of Equations (12) and (16) for higher initial heights results in *E_R_* > 100% and is thus not considered meaningful.

**Table 19:** Calculation of the theoretical spring constant *k_STFR_* and *k_h_* and of the theoretical damping *c_s_* and *c_h_.* (mean ± maximum error).

Initial Height *h* [mm]	*k_STFR_* [N/mm]	*k_h_* [N/mm]	*c_s_* [N·s/m]	*c_h_* [N·s/m]
25 ± 1	4.525 ± 0.089	13.688 ± 0.075	23.424 ± 0.352	7.730 ± 0.641
50 ± 1	4.918 ± 0.064	15.460 ± 0.060	24.692 ± 0.347	6.425 ± 0.394
75 ± 1	4.850 ± 0.064	17.272 ± 0.076	25.776 ± 0.407	6.546 ± 0.490

Values for *t_P_*_0_ and *G_max_* were taken from Table 5, values for *v*_0_ were taken from Table 12 (average model). Calculations are given in File S15.

**Table 20:** Formulae for initial height currently implemented in the STFR software 1.0 and suggested modified formulae.

Currently Implemented Formulae (VST and STFR)	Suggested Improved Formulae for the STFR
$h=\frac{g}{2}\cdot {T_{0}}^{2}$,	$h=-\frac{2\cdot l^{2}}{g\cdot {T_{0}}^{2}}+\frac{l}{g\cdot {T_{0}}^{2}}\cdot \sqrt{4\cdot l^{2}+2\cdot g^{2}\cdot {T_{0}}^{4}},$
$\frac{∆h}{h}=2\cdot \frac{∆T_{0}}{T_{0}}.$	$\begin{array}{c} ∆h=\left|\frac{4\cdot l}{g\cdot {T_{0}}^{2}}-\frac{8\cdot l^{2}+2\cdot g^{2}\cdot {T_{0}}^{4}}{g\cdot {T_{0}}^{2}}\cdot \frac{\sqrt{4\cdot l^{2}+2\cdot g^{2}\cdot {T_{0}}^{4}}}{4\cdot l^{2}+2\cdot g^{2}\cdot {T_{0}}^{4}}\right|\cdot ∆l\\ \;\;\;\;\;\;\;\;\;\;\;\;\;\;\;\;\;\;\;\;\;\;\;\;\;\;\;\;\;\;\;\;\;+\left|\frac{4\cdot l^{2}}{g\cdot {T_{0}}^{3}}-\frac{8\cdot l^{3}}{g\cdot {T_{0}}^{3}}\cdot \frac{\sqrt{4\cdot l^{2}+2\cdot g^{2}\cdot {T_{0}}^{4}}}{4\cdot l^{2}+2\cdot g^{2}\cdot {T_{0}}^{4}}\right|\cdot ∆T_{0}. \end{array}$

**Table 21:** Formulae for impact velocity and penetration depth currently implemented in the STFR software and suggested modified formulae.

Currently Implemented Formulae (VST and STFR)	Suggested Improved Formulae for the STFR
$v_{0}\;=\;g\cdot T_{0}$, $\frac{∆v_{0}}{\left|v_{0}\right|}=\frac{∆T_{0}}{T_{0}}.$	$v_{0}=\left(-\right)g\cdot T_{0},$ $\frac{∆v_{0}}{\left|v_{0}\right|}=\frac{∆T_{0}}{T_{0}}.$
$D=g\cdot t_{P0}\cdot \left(T_{0}-\frac{G_{max}\cdot t_{P0}}{6}\right),$	$D=\frac{t_{P0}}{6}\cdot \left(2\cdot v_{0}-g\cdot G_{max}\cdot t_{P0}\right),$
$\begin{array}{l} ∆D=g\cdot \left|T_{0}-\frac{G_{max}\cdot t_{P0}}{3}\right|\cdot ∆t_{P0}+g\cdot t_{P0}\\ \;\;\;\;\;\;\;\;\;\;\;\;\;\;\;\;\;\;\;\;\;\;\;\;\;\;\;\;\cdot ∆T_{0}+\frac{{t_{P0}}^{2}}{6}\cdot g\cdot ∆G_{max}. \end{array}$	$∆D=\frac{\left|v_{0}-G_{max}\cdot g\cdot t_{P0}\right|}{3}\cdot ∆t_{P0}+\frac{t_{P0}}{3}\cdot ∆v_{0}+\frac{{t_{P0}}^{2}}{6}\cdot g\cdot ∆G_{max}$ with $v_{0}=-\left(1-\frac{h^{2}}{2\cdot l^{2}}\right)\cdot g\cdot T_{0},$$\frac{∆v_{0}}{\left|v_{0}\right|}=\frac{2\cdot h^{2}}{2\cdot l^{2}-h^{2}}\cdot \left(\frac{∆h}{h}+\frac{∆l}{l}\right)+\frac{∆T_{0}}{T_{0}}.$

**Table 22:** Formulae for Young’s Modulus currently implemented in the STFR software and suggested modified formulae.

Suggested Revised Formulae for the STFR
$E^{*}=\frac{3}{4}\cdot \frac{m\cdot g\cdot G_{max}}{\sqrt{R}\cdot \sqrt{{\left|D\right|}^{3}}},\;\;\frac{∆E^{*}}{E^{*}}=\frac{∆m}{m}+\frac{∆G_{max}}{G_{max}}+\frac{1}{2}\cdot \frac{∆R}{R}+\frac{3}{2}\frac{∆D}{\left|D\right|}.$

**Table 23:** Formulae for spring constant currently implemented in the STFR software and suggested modified formulae.

Currently Implemented Formulae (VST and STFR)	Suggested Improved Formulae for the STFR
$K=\frac{{G_{max}}^{2}\cdot m}{{T_{0}}^{2}},\;\;\frac{∆K}{K}=2\cdot \frac{∆G_{max}}{G_{max}}+2\cdot \frac{∆T_{0}}{T_{0}}+\frac{∆m}{m}.$	
No formula available	$k_{h}=\left(\frac{a_{max}\cdot \left(a_{max}-g\right)}{{v_{0}}^{2}}+\frac{2\cdot \left(a_{max}-g\right)}{t_{P0}\cdot v_{0}}+\frac{2}{{t_{P0}}^{2}}-\frac{{v_{0}}^{2}}{l^{2}}\right)\cdot m,$ $\begin{array}{l} ∆k_{h}=\left|\frac{G_{max}\cdot g^{2}\cdot \left(G_{max}-1\right)}{{v_{0}}^{2}}+\frac{2\cdot g\cdot \left(G_{max}-1\right)}{t_{P0}\cdot v_{0}}+\frac{2}{{t_{P0}}^{2}}-\frac{{v_{0}}^{2}}{l^{2}}\right|\cdot ∆m+m\\ \;\;\;\;\;\;\;\;\;\;\;\;\;\;\;\;\;\;\;\;\;\;\;\;\;\;\;\cdot \left|\frac{2\cdot G_{max}-1}{{v_{0}}^{2}}\cdot g+\frac{2}{t_{P0}\cdot v_{0}}\right|\cdot g\cdot ∆G_{max}+\frac{2\cdot m}{{t_{P0}}^{2}}\\ \;\;\;\;\;\;\;\;\;\;\;\;\;\;\;\;\;\;\;\;\;\;\;\;\;\;\;\cdot \left|\frac{G_{max}-1}{v_{0}}\cdot g+\frac{2}{t_{P0}}\right|\cdot ∆t_{P0}+2\cdot m\\ \;\;\;\;\;\;\;\;\;\;\;\;\;\;\;\;\;\;\;\;\;\;\;\;\;\;\;\cdot \left|\frac{G_{max}\cdot g^{2}\cdot \left(G_{max}-1\right)}{{v_{0}}^{3}}+\frac{G_{max}-1}{t_{P0}\cdot {v_{0}}^{2}}\cdot g+\frac{v_{0}}{l^{2}}\right|\cdot ∆v_{0}\\ \;\;\;\;\;\;\;\;\;\;\;\;\;\;\;\;\;\;\;\;\;\;\;\;\;\;\;+\frac{2\cdot {v_{0}}^{2}\cdot m}{l^{3}}\cdot ∆l. \end{array}$

**Table 24:** Suggested new formulae for damping constants.

Suggested New Formulae for the STFR
$c_{s}=-\frac{G_{max}+1}{v_{0}}\cdot g\cdot m,$ $∆c_{s}=g\cdot \left|\frac{m}{v_{0}}\right|\cdot ∆G_{max}+\left|\frac{G_{max}+1}{{v_{0}}^{2}}\right|\cdot g\cdot m\cdot ∆v_{0}+\left\lceil \frac{G_{max}+1}{v_{0}}\right\rceil \cdot g\cdot ∆m.$
$c_{h}=\left(\frac{G_{max}-1}{v_{0}}\cdot g+\frac{2}{t_{P0}}\right)\cdot m,$ $∆c_{h}=\left|\frac{G_{max}-1}{v_{0}}\cdot g+\frac{2}{t_{P0}}\right|\cdot ∆m+\frac{m}{\left|v_{0}\right|}\cdot g\cdot ∆G_{max}+\frac{2\cdot m}{{t_{P0}}^{2}}\cdot ∆t_{P0}+\frac{m\cdot \left(G_{max}-1\right)}{{v_{0}}^{2}}\cdot g\cdot ∆v_{0}.$

**Table 25:** Formulae for calculation of Energy Recovery currently implemented in the STFR software and suggested modified formulae.

Currently Implemented Formulae (VST and STFR)	Suggested Improved Formulae for the STFR
$E_{R}={\left(\frac{T_{1}}{T_{0}}\right)}^{2},$ $\frac{∆E_{R}}{E_{R}}=2\cdot \left(\frac{{∆T}_{0}}{T_{0}}+\frac{∆T_{1}}{T_{1}}\right).$	For 25 mm ± 1 mm: $E_{R}\;=\;{\left(\frac{T_{1}}{T_{0}}\right)}^{2},\;\;\;\;\;\;\;\;\frac{∆E_{R}}{E_{R}}=2\cdot \left(\frac{{∆T}_{0}}{T_{0}}+\frac{∆T_{1}}{T_{1}}\right).$
	For 50 mm ± 1 mm and 75 mm ± 1 mm: $v_{1}=-\frac{g\cdot G_{max}\cdot \left(d_{0}-t_{P0}\right)}{2}-\frac{3\cdot D}{d_{0}-t_{P0}},$ $\begin{array}{c} ∆v_{1}=\frac{d_{0}-t_{P0}}{2}\cdot g\cdot ∆G_{max}+\frac{3}{d_{0}-t_{P0}}\cdot ∆h_{min}+\frac{1}{2}\\ \;\;\;\;\;\;\;\;\;\;\;\;\;\;\;\;\;\;\;\;\;\;\;\;\;\;\;\;\;\;\;\;\cdot \left|\frac{G_{max}\cdot g\cdot {\left(d_{0}-t_{P0}\right)}^{2}-6\cdot h_{min}}{{\left(d_{0}-t_{P0}\right)}^{2}}\right|\\ \cdot \left(∆d_{0}+∆t_{P0}\right) \end{array}$ with $E_{r}=\frac{{v_{1}}^{2}}{2\cdot g\cdot h},\;\;\;\;\;\;\frac{∆E_{r}}{E_{r}}=\frac{∆h}{h}+2\cdot \frac{∆v_{1}}{v_{1}}$.

**Table 26:** Suggested formulae for resonance frequency, which remain unchanged.

Currently Implemented and Retained Formulae for the VST and STFR
$f_{n}=\frac{1}{2\cdot d_{0}},\;\;\;\;\frac{∆f_{n}}{f_{n}}=\frac{∆d_{0}}{d_{0}}.$

## Data Availability

Data are contained in the Supplementary Files.

## Supplementary Materials

The following supporting information can be downloaded at: https://www.mdpi.com/article/10.3390/s25206307/s1, File S1: SURF_TST_29012025.xlsx; File S2: Derivations_of_the_formulae_2.pdf; File S3: comparison_different_start_height.mcdx; File S4: monotony_study.mcdx; File S5: reference_values_T0.mcdx; File S6: evaluation_28102004.xlsx; File S7: contour_of_the_sphere.ggb; File S8: calculate_the_ratio_vab_0_to_60_degrees.mcdx; File S9: calculate_h_hdelta.mcdx; File S10: calculate_v0_v0delta.mcdx; File S11: calculate_D_various_ways.mcdx; File S12: calculate_v1_v1delta.mcdx; File S13: calculate_hmax_hmaxdelta.mcdx; File S14: calculate_ER_various_ways.mcdx; File S15: calculate_k_c_various_ways.mcdx; File S16: comparison_spring_constant_damping_constant.xlsx; File S17: Methodology_revised 2025.pdf.

## Abbreviations

The following abbreviations are used in this manuscript:

|*x*|	Absolute amount (modulus) of *x*
*∂f/∂x*	Partial derivative of the function f with respect to the variable *x*
*∂f/∂y*	Partial derivative of the function f with respect to the variable *y*
Δ	Difference, error
*2·T*_1_	Duration of the second free-flight phase, [s]
*a*	Half major axis length of an ellipse, [mm]
apparent outline	The apparent outline is the image of the true outline under a projection.
*b*	Half minor axis length of an ellipse, [mm]
*cf*	Conversion factor
*c_h_*	Damping constant *c_h_* for deeper penetration, [N·s·m^−1^]
*c_s_*	Damping constant near the specimen’s surface, [N·s·m^−1^]
*D = h_min_*	Maximum penetration depth of the specimen, [mm]
*d_0_*	Duration of the first contact phase of the sphere with the specimen surface, [ms]
*E**	Young’s Modulus, [kPa]
*E_R_*	Energy restitution, %
*f = f(x,y)*	Function *f* of two variables *x* and *y*
*f_n_*	Resonance frequency, [Hz]
*g*	*g* = 9.81 m·s^−2^
*G_max_*	Factor for calculating the peak acceleration, [g-units]
*h*	Initial height, [mm]
*h_max_*	First rebound: maximum height, [mm]
*K, k*	Spring constant, [N·mm^−1^]
*k_h_*	Spring constant (power series calculation), [N·mm^−1^]
*k_STFR_*	Spring constant calculated by STFR, [N·mm^−1^]
*l*	Length of the rod, [mm]
*n*	Distance between the eye point and the image plane, [mm]
*pd*	Deviation, %
rad	Radiant
*SE*	Standard error
*T_o_*	Duration from release of the sphere to first impact, [s]
*t_P_*_0_	Duration from first sphere-specimen contact phase to the extremum of acceleration, [ms]
true outline	The true outline (contour, contour generator) is the set of all surface points where a projection ray touches the surface.
*v*_0_	Velocity at first impact of the sphere, [m·s^−1^]
*v_ab_*	Ratio of the major axis length to the minor axis length
*x*	Height, [mm]
*φ*	Angle
