# Supplementary material for: Image-Analysis-Based Validation of the Mathematical Framework for the Representation of the Travel of an Accelerometer-Based Texture Testing Device
Source: Sensors (Basel). 2025 Oct 12;25(20):6307. doi: 10.3390/s25206307 (PMC12567729; doi:10.3390/s25206307)
Supplement: Supplementary file 1 [file sensors-25-06307-s001.zip › File S2 Derivations_of_the_formulae_2.pdf]

Harald Paulsen

Supplementary file

Derivations of the formulas 2

In this document, the derivations are sorted by chapter number. To make them easier to follow, all intermediate steps are generally listed. Formulas that originate from the article retain their numbering.

Chapter 2.2.1

Formula 1

In this section, the relative deviation of the deflections at different starting positions is described mathematically.

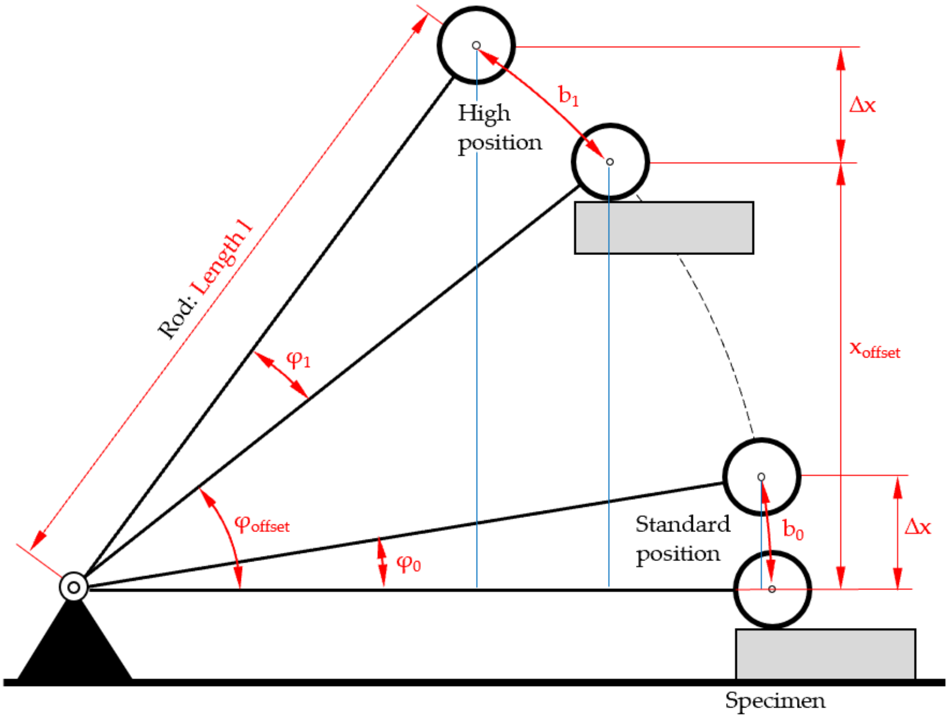

The deflection in the standard position is described by the angle φ_0

$$sin(\varphi_0) = \frac{\Delta x}{l}$$

$$\varphi_0 = arcsin\frac{\Delta x}{l}$$

For the position raised by x_{offset}, this results in a deflection φ_{offset}

$$sin(\varphi_{offset}) = \frac{x_{offset}}{l}$$

$$\varphi_{offset} = arcsin\frac{x_{offset}}{l}$$

For the high position, the total deflection is calculated as

$$sin(\varphi_1 + \varphi_{offset}) = \frac{\Delta x + x_{offset}}{l}$$

This gives the deflection φ_1 in the high position

$$\varphi_1 = arcsin\frac{\Delta x + x_{offset}}{l} - \varphi_{offset}$$

$$\varphi_1 = arcsin\frac{\Delta x + x_{offset}}{l} - arcsin\frac{x_{offset}}{l}$$

Result:

Relative deviation of the deflection φ_1 from the deflection φ_0 in the standard position:

$$\begin{aligned} rd_{\varphi} = 100 \cdot \frac{\varphi_1 - \varphi_0}{\varphi_0} &= 100 \cdot \frac{arcsin\frac{\Delta x + x_{offset}}{l} - arcsin\frac{x_{offset}}{l} - arcsin\frac{\Delta x}{l}}{arcsin\frac{\Delta x}{l}} \\ &= 100 \cdot \left(\frac{arcsin\frac{\Delta x + x_{offset}}{l} - arcsin\frac{x_{offset}}{l}}{arcsin\frac{\Delta x}{l}} - 1 \right) \end{aligned} \tag{1}$$

Chapter 2.2.3

This section examines the conditions under which the contour of a sphere is approximately circular. This question is of particular importance because the measurement mark applied to the STFR could not be used for the video evaluation and instead the contour of the sphere was used for the analysis.

We use the following German technical terms:

- Zentralprojektion: central projection
- Augpunkt A: center of projection
- Bildebene: image plane
- Augdistanz n: distance of the center of projection to the image plane.
- Sehstrahl: Connecting line of the center of projection with another point
- Kontur, wahrer Umriss: Contour, true outline
- Umriss, scheinbarer Umriss: Outline, apparent outline
- Bildpunkt, Zentralriss: picture point
- Hauptsehstrahl: Line through the center of projection that is perpendicular to the image plane
- Verschwindungsebene: Plane parallel to the image plane; runs through the center of projection.

For the mathematical-geometric analysis, we specify the following data:

- Radius R of the sphere
- Augdistanz n (>0); distance of the center of projection A (Augpunkt) to the image plane. In the impact position, the Augdistanz is also the distance from A to the center of the sphere M_φ.
- Angle of inclination ν between the line through the center of the sphere M_φ and the Hauptsehstrahl.

The information described above is shown in the following diagram:

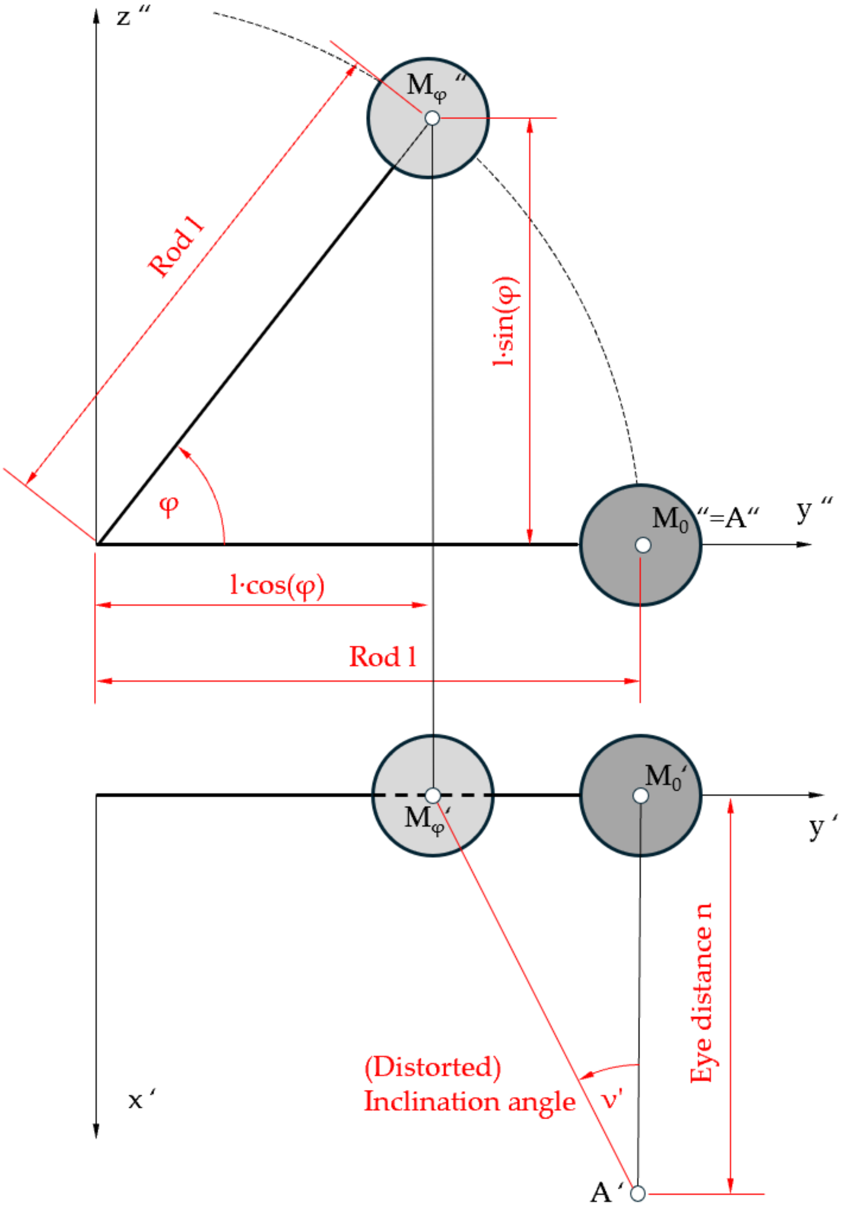

Step 1: Determining the angle of inclination v

Although the aim is to obtain a coordinate-independent representation of the angle v, we use a local coordinate system in the derivation.

$$A = \begin{pmatrix} n \\ l \\ 0 \end{pmatrix}$$

$$M_0 = \begin{pmatrix} 0 \\ l \\ 0 \end{pmatrix}$$

$$M_\varphi = \begin{pmatrix} 0 \\ l \cdot cos(\varphi) \\ l \cdot sin(\varphi) \end{pmatrix}$$

$$cos(v) = \frac{\overrightarrow{AM_0} \cdot \overrightarrow{AM_\varphi}}{\left|\overrightarrow{AM_0}\right| \cdot \left|\overrightarrow{AM_\varphi}\right|} = \frac{\begin{pmatrix} -n \\ 0 \\ 0 \end{pmatrix} \cdot \begin{pmatrix} -n \\ l \cdot \cos(\varphi) - l \\ l \cdot sin(\varphi) \end{pmatrix}}{n \cdot \sqrt{n^2 + (l \cdot cos(\varphi) - l)^2 + l^2 \cdot sin^2(\varphi)}}$$
$$= \frac{n^2}{n \cdot \sqrt{n^2 + l^2 \cdot cos^2(\varphi) - 2 \cdot l^2 \cdot cos(\varphi) + l^2 + l^2 \cdot sin^2(\varphi)}}$$
$$= \frac{n}{\sqrt{n^2 - 2 \cdot l^2 \cdot cos(\varphi) + 2 \cdot l^2}}$$

This results in the following for the angle v:

$$cos(v) = \frac{n}{\sqrt{n^2 - 2 \cdot l^2 \cdot cos(\varphi) + 2 \cdot l^2}}$$

Step 2: Determining the mapping equation of the central projection

For the following calculation, we use a coordinate system adapted to the task. In this, the Augpunkt A and the center of the sphere M_φ have the following coordinates.

$$A = \begin{pmatrix} 0 \\ 0 \\ n \end{pmatrix}$$

$$M_\varphi = \begin{pmatrix} 0 \\ y_\varphi \\ 0 \end{pmatrix} = \begin{pmatrix} 0 \\ -n \cdot tan(v) \\ 0 \end{pmatrix}$$

For the derivation, we need a mapping equation that assigns a perspective view Q^c in the Bildebene to each point Q in space (with the exception of the Augpunkt and the points of the Verschwindungsebene).

For the central projection of any point $Q = \begin{pmatrix} x \\ y \\ z \end{pmatrix}$, the Sehstrahl passing through A is parameterised as follows

$$X(\lambda) = A + \lambda \cdot \overrightarrow{AQ} = \begin{pmatrix} 0 \\ 0 \\ n \end{pmatrix} + \lambda \cdot \begin{pmatrix} x \\ y \\ z - n \end{pmatrix}$$

When projecting onto the ground plane, the vanishing z-coordinate gives

$$0 = n + \lambda \cdot (z - n)$$

and therefore due to

$$\lambda = -\frac{n}{z - n}$$

$$Q^c = X\left(\lambda = -\frac{n}{z-n}\right) = \begin{pmatrix} 0 \\ 0 \\ n \end{pmatrix} - \frac{n}{z-n} \cdot \begin{pmatrix} x \\ y \\ z - n \end{pmatrix} = -n \cdot \begin{pmatrix} \frac{x}{z-n} \\ \frac{y}{z-n} \\ 0 \end{pmatrix}$$

This is the mapping equation:

$$Q = \begin{pmatrix} x \\ y \\ z \end{pmatrix} \rightarrow Q^c = -n \cdot \begin{pmatrix} \frac{x}{z-n} \\ \frac{y}{z-n} \\ 0 \end{pmatrix}$$

Step 3: Calculation of the main axis lengths of the image ellipse

The following calculations are illustrated by the construction (view from the front and view from above). While the eye point lies in front of the vertical Bildebene in the STFR setup, we consider a situation rotated by 90°. Since the measuring sphere moves on a circular path, all positions of the measuring sphere are predefined. The deflection φ of the sphere is already taken into account in the coordinate representation of its center M_φ.

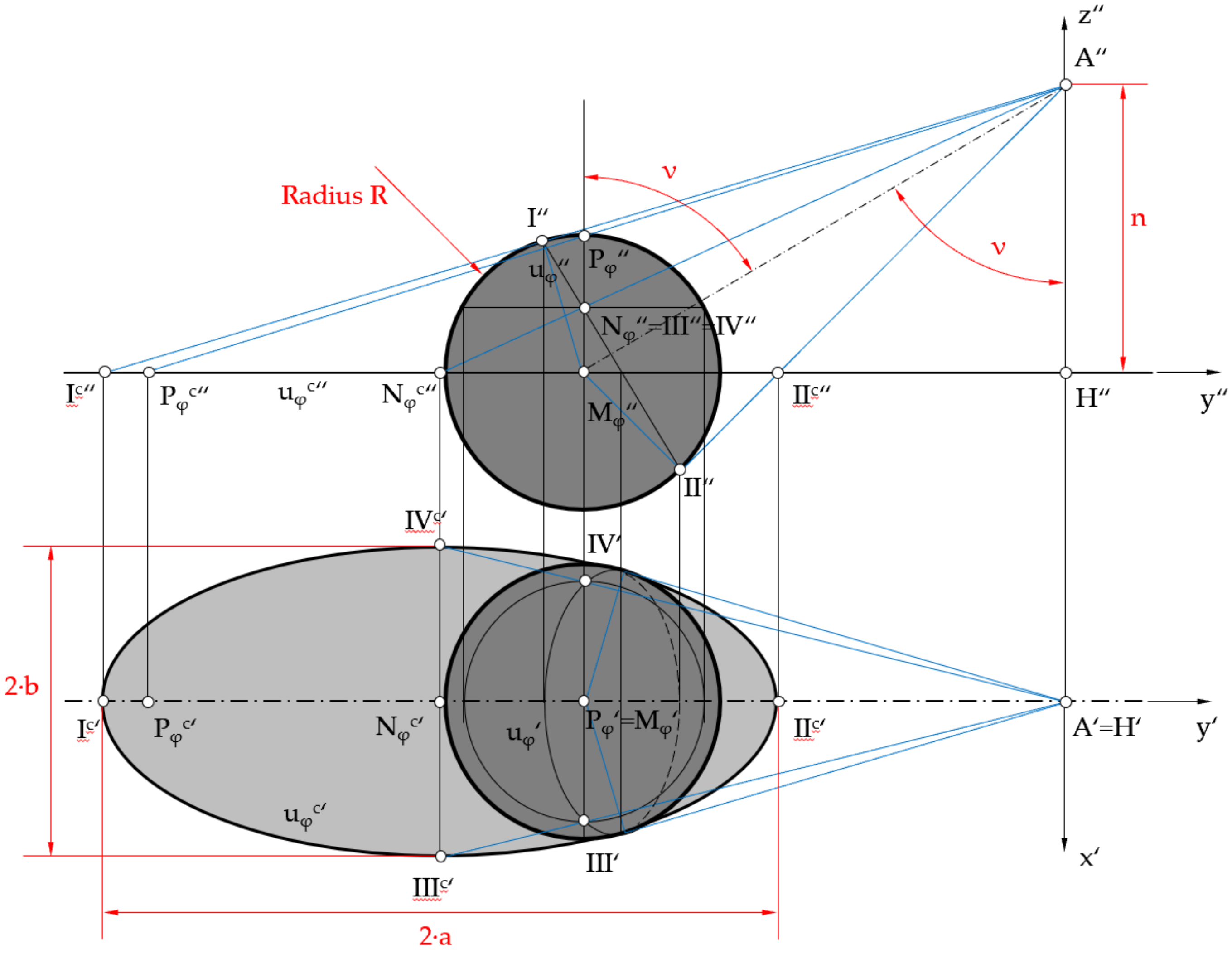

Because the Verschwindungsebene does not intersect or touch the sphere, the contour of the sphere is an ellipse with half the axis lengths a and b. It is to be investigated under which conditions this ellipse can be assumed to be approximately a circle.

The ratio $v_{ab}=a:b$ now only depends on the Augdistanz n and the angle of inclination ν. Therefore, a table is created in which the relationship between n, ν and v_{ab} is listed. It is essential for the derivation that one can restrict oneself to a simple list due to symmetry properties.

In addition, parallel displacement of the Bildebene does not change the ratio v_{ab}, which is why the Bildebene can be placed through the center of the sphere in favour of a simpler calculation.

- Determination of the main axis length 2·a

The contact condition applies for the spherical points I and II (x=0) and provides an equation in which y and z occur.

$$\overrightarrow{M_\varphi I} \cdot \overrightarrow{AI} = 0 \text{ and } \overrightarrow{M_\varphi II} \cdot \overrightarrow{AII} = 0$$

Using the coordinates $\begin{pmatrix} 0 \\ y \\ z \end{pmatrix}$ - initially for both points I and II - the result is:

$$\begin{pmatrix} 0 \\ y - y_\varphi \\ z \end{pmatrix} \cdot \begin{pmatrix} 0 \\ y \\ z - n \end{pmatrix} = 0$$

$$(y - y_\varphi) \cdot y + z \cdot (z - n) = 0$$

I and II lie on the measuring sphere. This gives us a second equation in y and z:

$$\left|\overrightarrow{M_\varphi I}\right| = R \text{ and } \left|\overrightarrow{M_\varphi II}\right| = R$$

$$(y - y_\varphi)^2 + z^2 = R^2$$

The two equations form a non-linear system of equations with two equations in two unknowns y and z.

$$y^2 - y_\varphi \cdot y + z^2 - z \cdot n = 0$$

$$y^2 - 2 \cdot y_\varphi \cdot y + {y_\varphi}^2 + z^2 = R^2$$

The quadratic terms with y^2 and z^2 are eliminated first. The subtraction of both equations results in

$$y_\varphi \cdot y - {y_\varphi}^2 - z \cdot n = -R^2$$

and after transformations we get

$$z = \frac{y_\varphi \cdot y - {y_\varphi}^2 + R^2}{n} = \frac{y_\varphi}{n} \cdot y + \frac{R^2 - {y_\varphi}^2}{n}$$

If this equation is considered in isolation in the y-z plane, then the equation describes the polar line of A.

The second equation of the system of equations is multiplied by n^2

$$y^2 \cdot n^2 - 2 \cdot y_\varphi \cdot y \cdot n^2 + {y_\varphi}^2 \cdot n^2 + z^2 \cdot n^2 = R^2 \cdot n^2$$

and in this equation z is replaced by

$$z = \frac{y_\varphi \cdot y - {y_\varphi}^2 + R^2}{n}$$

.

This leads to a quadratic equation in y:

$$y^2 \cdot n^2 - 2 \cdot y_\varphi \cdot y \cdot n^2 + {y_\varphi}^2 \cdot n^2 + \left(\frac{y_\varphi \cdot y - {y_\varphi}^2 + R^2}{n}\right)^2 \cdot n^2 = R^2 \cdot n^2$$

After the simplifications

$$y^2 \cdot n^2 - 2 \cdot y_\varphi \cdot y \cdot n^2 + {y_\varphi}^2 \cdot n^2 + \left(y_\varphi \cdot y - {y_\varphi}^2 + R^2\right)^2 = R^2 \cdot n^2$$

$$y^2 \cdot n^2 - 2 \cdot y_\varphi \cdot y \cdot n^2 + \left(y_\varphi \cdot y + R^2 - {y_\varphi}^2\right)^2 - \left(R^2 - {y_\varphi}^2\right) \cdot n^2 = 0$$

$$y^2 \cdot n^2 - 2 \cdot y_\varphi \cdot y \cdot n^2 + {y_\varphi}^2 \cdot y^2 + 2 \cdot y_\varphi \cdot y \cdot \left(R^2 - {y_\varphi}^2\right) + \left(R^2 - {y_\varphi}^2\right)^2 - \left(R^2 - {y_\varphi}^2\right) \cdot n^2 = 0$$

$$y^2 \cdot n^2 + {y_\varphi}^2 \cdot y^2 - 2 \cdot y_\varphi \cdot y \cdot n^2 + 2 \cdot y_\varphi \cdot y \cdot \left(R^2 - {y_\varphi}^2\right) + \left(R^2 - {y_\varphi}^2\right)^2 - \left(R^2 - {y_\varphi}^2\right) \cdot n^2 = 0$$

$$y^2 \cdot \left(n^2 + {y_\varphi}^2\right) - 2 \cdot y \cdot y_\varphi \cdot \left(n^2 - R^2 + {y_\varphi}^2\right) + \left(R^2 - {y_\varphi}^2\right)^2 - \left(R^2 - {y_\varphi}^2\right) \cdot n^2 = 0$$

$$y^2 \cdot \left(n^2 + {y_\varphi}^2\right) + 2 \cdot y \cdot y_\varphi \cdot \left(R^2 - {y_\varphi}^2 - n^2\right) + \left(R^2 - {y_\varphi}^2\right) \cdot \left(R^2 - {y_\varphi}^2 - n^2\right) = 0$$

$$y^2 + 2 \cdot y \cdot y_\varphi \cdot \frac{R^2 - {y_\varphi}^2 - n^2}{n^2 + {y_\varphi}^2} + \left(R^2 - {y_\varphi}^2\right) \cdot \frac{R^2 - {y_\varphi}^2 - n^2}{n^2 + {y_\varphi}^2} = 0$$

$$y_{1,2} = -y_\varphi \cdot \frac{R^2 - {y_\varphi}^2 - n^2}{n^2 + {y_\varphi}^2} \pm \sqrt{{y_\varphi}^2 \cdot \frac{\left(R^2 - {y_\varphi}^2 - n^2\right)^2}{\left(n^2 + {y_\varphi}^2\right)^2} - \left(R^2 - {y_\varphi}^2\right) \cdot \frac{R^2 - {y_\varphi}^2 - n^2}{n^2 + {y_\varphi}^2}}$$

$$y_{1,2} = -y_\varphi \cdot \frac{R^2 - {y_\varphi}^2 - n^2}{n^2 + {y_\varphi}^2} \pm \sqrt{{y_\varphi}^2 \cdot \frac{\left(R^2 - {y_\varphi}^2 - n^2\right)^2}{\left(n^2 + {y_\varphi}^2\right)^2} - \frac{\left(R^2 - {y_\varphi}^2\right) \cdot \left(R^2 - {y_\varphi}^2 - n^2\right)}{\left(n^2 + {y_\varphi}^2\right)}}$$

$$y_{1,2} = -y_\varphi \cdot \frac{R^2 - {y_\varphi}^2 - n^2}{n^2 + {y_\varphi}^2} \pm \sqrt{{y_\varphi}^2 \cdot \frac{\left(R^2 - {y_\varphi}^2 - n^2\right)^2}{\left(n^2 + {y_\varphi}^2\right)^2} - \frac{\left(R^2 - {y_\varphi}^2\right) \cdot \left(R^2 - {y_\varphi}^2 - n^2\right) \cdot \left(n^2 + {y_\varphi}^2\right)}{\left(n^2 + {y_\varphi}^2\right)^2}}$$

we obtain the solutions of the quadratic equation as

$$y_{1,2} = -y_\varphi \cdot \frac{R^2 - {y_\varphi}^2 - n^2}{n^2 + {y_\varphi}^2} \pm \frac{1}{n^2 + {y_\varphi}^2} \cdot \sqrt{{y_\varphi}^2 \cdot \left(R^2 - {y_\varphi}^2 - n^2\right)^2 - \left(R^2 - {y_\varphi}^2\right) \cdot \left(R^2 - {y_\varphi}^2 - n^2\right) \cdot \left(n^2 + {y_\varphi}^2\right)}$$

This results in the following coordinates for points *I* and *II*:

$$I = \begin{pmatrix} 0 \\ y_1 \\ z_1 \end{pmatrix} \ and\ II = \begin{pmatrix} 0 \\ y_2 \\ z_2 \end{pmatrix}$$

$$y_{1,2} = -y_\varphi \cdot \frac{R^2 - {y_\varphi}^2 - n^2}{n^2 + {y_\varphi}^2} \pm \frac{1}{n^2 + {y_\varphi}^2} \cdot \sqrt{{y_\varphi}^2 \cdot \left(R^2 - {y_\varphi}^2 - n^2\right)^2 - \left(R^2 - {y_\varphi}^2\right) \cdot \left(R^2 - {y_\varphi}^2 - n^2\right) \cdot \left(n^2 + {y_\varphi}^2\right)}$$

$$z_{1,2} = \frac{y_\varphi}{n} \cdot y_{1,2} + \frac{R^2 - {y_\varphi}^2}{n}$$

- The perspective views I^c and II^c then result as

$$I^c = -n \cdot \begin{pmatrix} 0 \\ \dfrac{y_1}{z_1 - n} \\ 0 \end{pmatrix} \ and\ II^c = -n \cdot \begin{pmatrix} 0 \\ \dfrac{y_2}{z_2 - n} \\ 0 \end{pmatrix}$$

and half the length of the main axis *a* is then

$$a = a(n, v) = \frac{n}{2} \cdot \left| \frac{y_2}{z_2 - n} - \frac{y_1}{z_1 - n} \right|$$

- Determining the minor axis length 2·*b*

Using the perspective views I^c and II^c, the center N^c of the image ellipse is obtained due to

$$\frac{y_1 + y_2}{2} = \frac{1}{2} \cdot \left(-y_\varphi \cdot \frac{R^2 - {y_\varphi}^2 - n^2}{n^2 + {y_\varphi}^2} + \frac{1}{n^2 + {y_\varphi}^2} \cdot \sqrt{{y_\varphi}^2 \cdot \left(R^2 - {y_\varphi}^2 - n^2\right)^2 - \left(R^2 - {y_\varphi}^2\right) \cdot \left(R^2 - {y_\varphi}^2 - n^2\right) \cdot \left(n^2 + {y_\varphi}^2\right)} - y_\varphi \cdot \frac{R^2 - {y_\varphi}^2 - n^2}{n^2 + {y_\varphi}^2} - \frac{1}{n^2 + {y_\varphi}^2} \cdot \sqrt{{y_\varphi}^2 \cdot \left(R^2 - {y_\varphi}^2 - n^2\right)^2 - \left(R^2 - {y_\varphi}^2\right) \cdot \left(R^2 - {y_\varphi}^2 - n^2\right) \cdot \left(n^2 + {y_\varphi}^2\right)} \right) = -y_\varphi \cdot \frac{R^2 - {y_\varphi}^2 - n^2}{n^2 + {y_\varphi}^2}$$

as

$$N^c = \begin{pmatrix} 0 \\ -y_\varphi \cdot \dfrac{R^2 - {y_\varphi}^2 - n^2}{n^2 + {y_\varphi}^2} \\ 0 \end{pmatrix}$$

This means that the original point N is located on the Sehstrahl AN^c. AN^c can therefore be represented as follows:

$$X(\mu) = A + \mu \cdot \overrightarrow{AN^c} = \begin{pmatrix} 0 \\ 0 \\ n \end{pmatrix} + \mu \cdot \begin{pmatrix} 0 \\ -y_\varphi \cdot \dfrac{R^2 - {y_\varphi}^2 - n^2}{n^2 + {y_\varphi}^2} \\ -n \end{pmatrix}$$

$$y = -y_\varphi \cdot \mu \cdot \frac{R^2 - {y_\varphi}^2 - n^2}{n^2 + {y_\varphi}^2}$$

$$z = n - \mu \cdot n$$

N also lies on the second Bildebene that contains the polar of A. Therefore, N is determined as the intersection of the straight line $X(\mu)$ with the polar line

$$z \cdot n = y_\varphi \cdot y - {y_\varphi}^2 + R^2$$

which was mentioned in a previous section. From the last three formulas we obtain an equation in μ:

$$n \cdot (n - \mu \cdot n) = -{y_\varphi}^2 \cdot \mu \cdot \frac{R^2 - {y_\varphi}^2 - n^2}{n^2 + {y_\varphi}^2} - {y_\varphi}^2 + R^2$$

After the transformations

$$n^2 - \mu \cdot n^2 = -{y_\varphi}^2 \cdot \mu \cdot \frac{R^2 - {y_\varphi}^2 - n^2}{n^2 + {y_\varphi}^2} - {y_\varphi}^2 + R^2$$

$$y_\varphi{}^2 \cdot \mu \cdot \frac{R^2 - y_\varphi{}^2 - n^2}{n^2 + y_\varphi{}^2} - \mu \cdot n^2 = R^2 - y_\varphi{}^2 - n^2$$

$$\mu \cdot \left(y_\varphi{}^2 \cdot \frac{R^2 - y_\varphi{}^2 - n^2}{n^2 + y_\varphi{}^2} - n^2 \right) = R^2 - y_\varphi{}^2 - n^2$$

$$\mu \cdot \frac{y_\varphi{}^2 \cdot \left(R^2 - y_\varphi{}^2 - n^2\right) - n^2 \cdot \left(n^2 + y_\varphi{}^2\right)}{n^2 + y_\varphi{}^2} = R^2 - y_\varphi{}^2 - n^2$$

we get the solution

$$\mu = \frac{\left(R^2 - y_\varphi{}^2 - n^2\right) \cdot \left(n^2 + y_\varphi{}^2\right)}{y_\varphi{}^2 \cdot \left(R^2 - y_\varphi{}^2 - n^2\right) - n^2 \cdot \left(n^2 + y_\varphi{}^2\right)}$$

and therefore the coordinates of N as:

$$N = \begin{pmatrix} 0 \\ y_n \\ z_n \end{pmatrix} = X\left(\mu = \frac{\left(R^2 - y_\varphi{}^2 - n^2\right) \cdot \left(n^2 + y_\varphi{}^2\right)}{y_\varphi{}^2 \cdot \left(R^2 - y_\varphi{}^2 - n^2\right) - n^2 \cdot \left(n^2 + y_\varphi{}^2\right)} \right)$$
$$= \begin{pmatrix} 0 \\ 0 \\ n \end{pmatrix} + \frac{\left(R^2 - y_\varphi{}^2 - n^2\right) \cdot \left(n^2 + y_\varphi{}^2\right)}{y_\varphi{}^2 \cdot \left(R^2 - y_\varphi{}^2 - n^2\right) - n^2 \cdot \left(n^2 + y_\varphi{}^2\right)} \cdot \begin{pmatrix} 0 \\ -y_\varphi \cdot \dfrac{R^2 - y_\varphi{}^2 - n^2}{n^2 + y_\varphi{}^2} \\ -n \end{pmatrix}$$

- The points *III* and *IV* have the y and z coordinates in common with N. Therefore, they can be set as $I, II = \begin{pmatrix} x \\ y_n \\ z_n \end{pmatrix}$. Since both lie on the sphere the following applies with

$$x^2 + \left(y - y_\varphi\right)^2 + z^2 = R^2$$

for I and II special

$$x^2 + \left(y_n - y_\varphi\right)^2 + z_n{}^2 = R^2$$

and thus for x:

$$x_{1,2} = \pm\sqrt{R^2 - \left(y_n - y_\varphi\right)^2 - z_n{}^2}$$

III and *IV* therefore have the coordinates

$$III, VI = \begin{pmatrix} \pm\sqrt{R^2 - \left(y_n - y_\varphi\right)^2 - z_n{}^2} \\ y_n \\ z_n \end{pmatrix}$$

and their perspective views are

$$III^c = -n \cdot \begin{pmatrix} \frac{x_1}{z_n - n} \\ \frac{y_n}{z_n - n} \\ 0 \end{pmatrix} \text{ and } IV^c = -n \cdot \begin{pmatrix} \frac{x_2}{z_n - n} \\ \frac{y_n}{z_n - n} \\ 0 \end{pmatrix}$$

Due to the symmetry of the ellipse with respect to the y-axis, half the minor axis length is

$$b = -n \cdot \frac{x_1}{z_n - n}$$

- Deviation from N^c to M^c and P^c

Due to the position of the image plane, the center M of the sphere is also its perspective view M^c.

$$M = M^c = \begin{pmatrix} 0 \\ y_\varphi \\ 0 \end{pmatrix}$$

The deviation d_{NM} between N^c and M^c is calculated as:

$$d_{NM} = |\overrightarrow{NM}| = \left| y_\varphi - y_\varphi \cdot \frac{R^2 - {y_\varphi}^2 - n^2}{n^2 + {y_\varphi}^2} \right| = \left| y_\varphi \cdot \frac{2 \cdot (n^2 + {y_\varphi}^2) - R^2}{n^2 + {y_\varphi}^2} \right|$$

The point $P = \begin{pmatrix} 0 \\ y_\varphi \\ R \end{pmatrix}$ above the centre of the sphere M can be used as a marking point. Its perspective view P^c is calculated as follows.

$$P^c = -n \cdot \begin{pmatrix} \frac{0}{R - n} \\ \frac{y_\varphi}{R - n} \\ 0 \end{pmatrix} = \begin{pmatrix} 0 \\ \frac{-n \cdot y_\varphi}{R - n} \\ 0 \end{pmatrix}$$

The deviation d_{NP} between N^c and P^c is calculated as:

$$d_{NP} = |\overrightarrow{NP}| = \left| \frac{n \cdot y_\varphi}{R - n} - y_\varphi \cdot \frac{R^2 - {y_\varphi}^2 - n^2}{n^2 + {y_\varphi}^2} \right|$$

Step 4: Implementation in software to create a reference table

The formulas required were taken from the previous sections.

- n is preset as the camera parameter.
- The angle v is determined as follows:

$$v = arccos \frac{n}{\sqrt{n^2 - 2 \cdot l^2 \cdot cos(\varphi) + 2 \cdot l^2}}$$

- The coordinate y_φ is obtained as:

$$y_\varphi = -n \cdot tan(v)$$

- For the coordinates y_1 and z_1 as well as y_2 and z_2 we use:

$$y_{1,2} = -y_\varphi \cdot \frac{R^2 - {y_\varphi}^2 - n^2}{n^2 + {y_\varphi}^2} \pm \frac{1}{n^2 + {y_\varphi}^2} \cdot \sqrt{{y_\varphi}^2 \cdot \left(R^2 - {y_\varphi}^2 - n^2\right)^2 - \left(R^2 - {y_\varphi}^2\right) \cdot \left(R^2 - {y_\varphi}^2 - n^2\right) \cdot \left(n^2 + {y_\varphi}^2\right)}$$

$$z_{1,2} = \frac{y_\varphi}{n} \cdot y_{1,2} + \frac{R^2 - {y_\varphi}^2}{n}$$

- To calculate the half major axis length a, we use

$$a = \frac{n}{2} \cdot \left| \frac{y_2}{z_2 - n} - \frac{y_1}{z_1 - n} \right|$$

- The y and z coordinates of the original point N are

$$N = \begin{pmatrix} 0 \\ y_n \\ z_n \end{pmatrix} = X(\mu) = \begin{pmatrix} 0 \\ 0 \\ n \end{pmatrix} + \frac{\left(R^2 - {y_\varphi}^2 - n^2\right) \cdot \left(n^2 + {y_\varphi}^2\right)}{{y_\varphi}^2 \cdot \left(R^2 - {y_\varphi}^2 - n^2\right) - n^2 \cdot \left(n^2 + {y_\varphi}^2\right)} \cdot \begin{pmatrix} 0 \\ -y_\varphi \cdot \frac{R^2 - {y_\varphi}^2 - n^2}{n^2 + {y_\varphi}^2} \\ -n \end{pmatrix}$$

$$y_n = -\frac{y_\varphi \cdot (R^2 - y_\varphi{}^2 - n^2) \cdot (n^2 + y_\varphi{}^2)}{y_\varphi{}^2 \cdot (R^2 - y_\varphi{}^2 - n^2) - n^2 \cdot (n^2 + y_\varphi{}^2)} \cdot \frac{R^2 - y_\varphi{}^2 - n^2}{n^2 + y_\varphi{}^2}$$
$$= -\frac{y_\varphi \cdot (R^2 - y_\varphi{}^2 - n^2)^2}{y_\varphi{}^2 \cdot (R^2 - y_\varphi{}^2 - n^2) - n^2 \cdot (n^2 + y_\varphi{}^2)}$$
$$z_n = n\left(1 - \frac{(R^2 - y_\varphi{}^2 - n^2) \cdot (n^2 + y_\varphi{}^2)}{y_\varphi{}^2 \cdot (R^2 - y_\varphi{}^2 - n^2) - n^2 \cdot (n^2 + y_\varphi{}^2)}\right)$$

- Calculation of the half minor axis length *b*:

$$x_1 = \sqrt{R^2 - (y_n - y_\varphi)^2 - z_n{}^2}$$
$$b = -n \cdot \frac{x_1}{z_n - n}$$

- Calculation of the ratio $v_{ab}=a:b$

The actual implementation is done in PTC Mathcad Prime 11®. The following applies to the prototype of the STFR because *l*=0.170 m, *R*=0.015 m and *n*=0.35 m + 0.015 m = 0.365 m. The table values (Table S1) were determined in the File: Calculate_the_ratio_vab_0_to_90a.mcdx.

Table S1: The table shows the size of the half-axis lengths *a* and *b*, their ratio v_{ab} and the deviations from N^c to M^c or P^c as a function of the deflection φ.

φ [deg]	*a* [mm]	*b* [mm]	$v_{ab}=a:b$	d_{NM} [mm]	d_{NP} [mm]
0	15	15	1	0	0
5	15.0375	15	1.0025	29.6362	0.6606
10	15.0997	15	1.0066	59.2162	1.3197
15	15.1860	15	1.0124	88.6840	1.9758
20	15.2957	15	1.0197	117.9836	2.6275
25	15.4277	15	1.0285	147.0595	3.2733
30	15.5806	15	1.0387	175.8565	3.9118
35	15.7529	15	1.0502	204.3198	4.5418
40	15.9429	15	1.0629	232.3954	5.1620
45	16.1487	15	1.0766	260.0298	5.7712
50	16.3684	15	1.0912	287.1703	6.3683
55	16.6000	15	1.1067	313.7653	6.9521
60	16.8413	15	1.228	339.7641	7.522

Step 5: Interpretation of the results

For measuring heights of 25 mm, 50 mm and 75 mm, the deflection φ can be calculated using the equation

$$.h = l \cdot sin(\varphi) \rightarrow \varphi = arcsin\frac{h}{l}$$

The deflections are shown in Table S2

Table S2: Maximum deflection φ of the measuring sphere as a function of the maximum drop height h

h [mm]	φ [deg]
25	8.4565
50	17.1045
75	26.1790

It can be concluded from Figure S1 that the function v_{ab} is monotonically increasing in the considered range [0°; 60°].

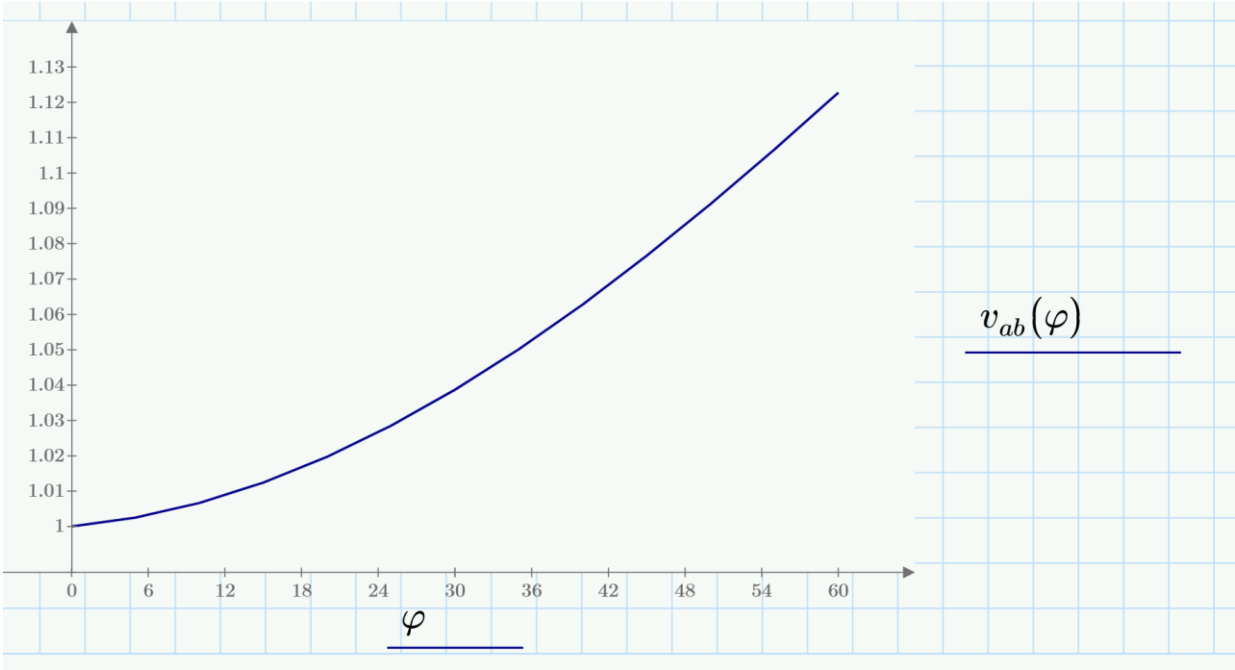

Figure S1: Representation of the function v_{ab}. The angle φ is given in degrees.

It can therefore be expected that for the maximum drop heights of 25 mm, 50 mm and 75 mm preset in the STFR for the video analysis, the contour of the sphere outline as a circle will lead to a maximum distortion v_{ab} that is less than 1.0387 = 103.87 % (due to the reference angle of 30°). Thus, assuming a minor axis length of $2{\cdot}b$ = 30 mm with a major axis length of $2{\cdot}a$ = 31.1612 mm, an absolute deviation of 0.5806 mm on both sides would be expected (see Figure S2).

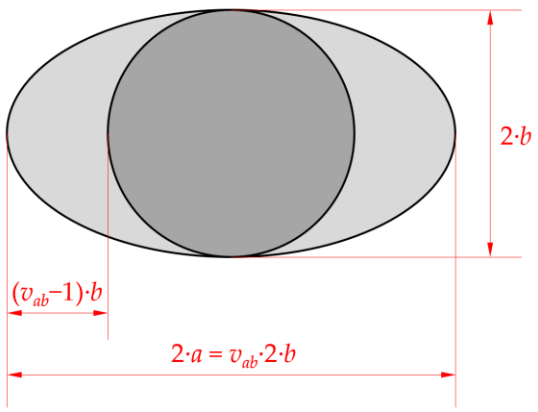

Figure S2: Effect of v_{ab} on the circular outline.

Smaller deflections φ lead to smaller deviations according to Table S1. It can therefore be assumed that the position data obtained from the video analysis becomes more accurate the closer you get to the point of impact.
